# Novel Miniature Membrane Active Lipopeptidomimetics against Planktonic and Biofilm Embedded Methicillin-Resistant *Staphylococcus aureus*

**DOI:** 10.1038/s41598-017-17234-z

**Published:** 2018-01-18

**Authors:** Seema Joshi, Sana Mumtaz, Jyotsna Singh, Santosh Pasha, Kasturi Mukhopadhyay

**Affiliations:** 1 Antimicrobial Research Laboratory, School of Environmental Sciences, Jawaharlal Nehru University, New Delhi, 110067 India; 2 Peptide Research Laboratory, CSIR-Institute of Genomics and Integrative Biology, Mall Road, New Delhi, 110007 India

## Abstract

Escalating multidrug resistance and highly evolved virulence mechanisms have aggravated the clinical menace of methicillin-resistant *Staphylococcus aureus* (MRSA) infections. Towards development of economically viable staphylocidal agents here we report eight structurally novel tryptophan-arginine template based peptidomimetics. Out of the designed molecules, three lipopeptidomimetics (S-6, S-7 and S-8) containing 12-amino dodecanoic acid exhibited cell selectivity and good to potent activity against clinically relevant pathogens MRSA, methicillin-resistant *Staphylococcus epidermidis* and vancomycin-resistant *Enterococcus faecium* (MIC: 1.4–22.7 μg/mL). Mechanistically, the active peptidomimetics dissipated membrane potential and caused massive permeabilization on MRSA concomitant with loss of viability. Against stationary phase MRSA under nutrient-depleted conditions, active peptidomimetics S-7 and S-8 achieved > 6 log reduction in viability upon 24 h incubation while both S-7 (at 226 μg/mL) and S-8 (at 28 μg/mL) also destroyed 48 h mature MRSA biofilm causing significant decrease in viability (p < 0.05). Encouragingly, most active peptidomimetic S-8 maintained efficacy against MRSA in presence of serum/plasma while exhibiting no increase in MIC over 17 serial passages at sub-MIC concentrations implying resistance development to be less likely. Therefore, we envisage that the current template warrants further optimization towards the development of cell selective peptidomimetics for the treatment of device associated MRSA infections.

## Introduction

Pan-resistant microbial pathogens refractory to current clinical antibiotics are spreading at an unprecedented rate^1^. Among human pathogens, methicillin-resistant *Staphylococcus aureus* (MRSA) is a high priority clinical threat^2^. Multidrug resistance and expression of multiple virulence factors have further exacerbated the clinical severity of MRSA infections in clinics as well as community settings^3^. Moreover, with the growing use of medical implants in clinics, almost 65–80% MRSA infections *in vivo* have been reported to be associated with biofilm formation. Biofilms are large agglomerations of bacterial cells encased in a self-produced matrix that exhibit substantial recalcitrance to antibiotic treatment^4^ (10 to 1000-fold more antibiotic concentration is required to eradicate biofilms). The recalcitrance of biofilms is multifactorial involving high localized inoculum, poor penetration of antibiotics to the core of biofilms, slow-growing nutrient-depleted bacterial populations and presence of persister cells^5,6^. Additionally, matrix formation allows the creation of a microenvironment which promotes resistance and tolerance development in microbes along with providing protection from host immune surveillance^7^. Therefore, a lot of efforts are being directed towards optimization of novel molecules/strategies to eradicate clinically relevant biofilms^8–10^. Recently, the consensus is emerging that unlike single target capturing antibiotics; membrane disruptive, dual targeting antibiotics such as oritavancin and daptomycin can eradicate slow-growing, persister cells and thus are more effective against biofilm embedded cells^11–13^. Among the alternatives to antibiotics with a novel membrane disruptive mode of action, cationic antimicrobial peptides (CAMPs) are lucrative candidates^14,15^.

CAMPs are small stretches of amino acid residues (16–50) which represent evolutionarily conserved components of host defense against invading microbes^16^. With an amphipathic arrangement of cationic and hydrophobic residues, CAMPs directly alter membrane permeability in microbes leading to perturbation of multiple cellular functions along with intracellular targeting to effectuate cell death^17^. The interest in developing CAMP based therapeutic agents also stems from the fact that these offer additional multifaceted functionalities including immune-modulation and synergy with conventional antibiotics with proven efficacy in animal infection models^18^. Although invaluable as a class of novel antibiotics, CAMPs are fraught with several limitations such as protease instability, high costs of production and inactivity under physiological conditions. Additionally, recently there have been alarming reports of resistance development^19^ against CAMPs and thus novel avenues based on their salient features are needed to be perused. Peptidomimetics serve as structurally simple and cost-effective surrogates of CAMPs owing to a smaller size while offering superior pharmacokinetic/pharmacodynamic parameters which may address the need for more efficacious systemic antimicrobial therapies. At present, there are several classes of investigational cationic antimicrobial peptidomimetics^20^ among which few molecules such as brilacidin, LTX-109 and XF-73 are under clinical trials for the treatment of Gram-positive bacterial infections^21,22^.

Previously, in our efforts to design cost-effective (short), resistance proof and membrane disruptive antibacterial peptides/peptidomimetics, we have explored the effect of increasing cationic charge by rational substitution of amino acids or modulation of hydrophobicity by using various N- and C-terminal unnatural modifications^23–26^. In our present work, we sought to develop short (5-mer), membrane disruptive, protease-stable peptidomimetics by incorporating unnatural moieties in the middle of the sequences to better fine tune hydrophobicity and thus activity/selectivity. Using these designed peptidomimetics we further explored the perspective that compared to conventional antibiotics membrane disruptive agents are better effective against biofilms. Towards this, we synthesized a focused series of eight novel synthetic molecules based on a previously defined tryptophan-arginine sequence template^27^ and screened their efficacy against a panel of clinically relevant Gram-positive bacterial strains. We further investigated the mechanism of action of active peptidomimetics against exponential phase planktonic MRSA cells using membrane depolarization/permeabilization and microscopy-based assays. Ultimately, we assessed kill kinetics of the active peptidomimetics against stationary phase MRSA in nutrient-depleted medium and extended the work against 48 h mature biofilms to evaluate their efficacy in biofilm model.

## Results and Discussion

### Design, synthesis and purification of peptidomimetics

**Figure 1:**
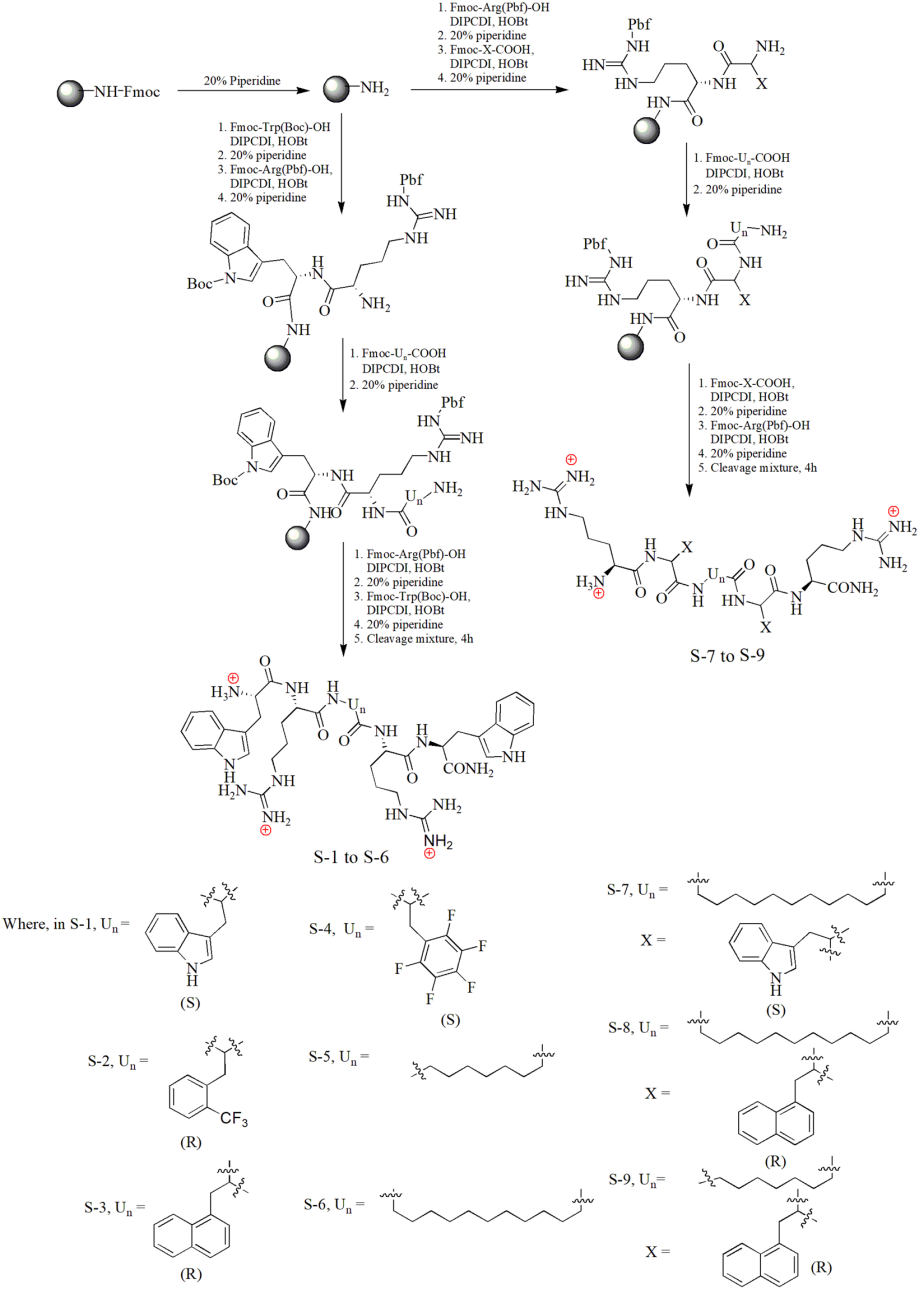
Synthetic scheme for designed peptidomimetics.

Based on the shortest pharmacophore for the design of CAMP mimics and various valuable structure-activity relationships a balance between cationic charge ( + 2 to + 9) and hydrophobicity has been established to be crucial to confer activity^28–30^. Therefore, keeping the minimum charge ( + 3), in the present work we modified hydrophobicity of the Trp-Arg rich pentapeptide template [Trp-Arg-Trp-Arg-Trp-CONH_2_ (S-1)] by incorporating various unnatural aromatic/aliphatic amino acids (U_n_) in the middle of the sequence [Trp-Arg-U_n_-Arg-Trp-CONH_2_] (Fig. 1). The high frequency of Trp and Arg residues in naturally occurring CAMPs^31^ (indolicidin, tritripticin and lactoferricin) and several promising Trp-Arg rich synthetic mimics^32,33^ prompted us to base our design on this template. To impart proteolytic stability while increasing hydrophobicity as U_n_ we incorporated unnatural aromatic amino acid residues such as 2-(trifluoromethyl)-D-phenylalanine (2- trifluoromethyl-D-Phe) in S-2, 3-(1-naphthyl)-D-alanine (D-1-Nal) in S-3 and 2-amino-3-pentafluoro phenyl-propionic acid (F_5_-Phe propionic acid) in S-4. We incorporated bulky aromatic amino acids as similar hydrophobic residues have previously been shown to improve antibacterial activity of resulting peptidomimetics^34–36^. The utility of enhanced hydrophobicity in promoting efficacy is not restricted to CAMP mimics only as clinical antibiotics like oritavancin, teicoplanin and telavancin are also reported to cause enhanced membrane permeability in microbes owing to better anchoring in bacterial cells due to the presence of appended hydrophobic side chains^37,38^. Based on RP-HPLC retention time which is a direct measure of hydrophobicity, S-1, S-2, and S-3 showed similar hydrophobicity while S-4 showed slight increase as compared to template sequence S-1 (Table 1). Previously for short peptides, N-terminal lipidation with long-chain fatty acids (C14 and C16) has been shown to impart excellent potency, however, most of such lipopeptides are difficult to optimize owing to associated toxicity^39^. Therefore, we incorporated small chain fatty acids (C8 and C12) in the middle of the sequence as U_n_ moiety to better fine tune hydrophobicity and thus, minimize toxicity. Specifically, linear short chain fatty acids 8-amino octanoic acid (8-AOA) and 12-amino dodecanoic acid (12-ADO) were incorporated as U_n_ in S-5 and S-6, respectively. Based on RP-HPLC retention time S-5 exhibited the lowest hydrophobicity among designed peptidomimetics while S-6 showed increased hydrophobicity compared to S-1 (Table 1).

**Table 1:** Name, composition, molecular mass, % purity and retention time of designed peptidomimetics.

Name	Composition	Molecular mass [M + H]^+^ (Da)		% purity^a^	Retention time^b^ (min)
		Calculated	Observed		
S-1	Trp-Arg-Trp-Arg-Trp-CONH_2_	888.4740	888.4759	97	23.1
S-2	Trp-Arg-(2-trifluoromethyl-D-Phe)-Arg-Trp-CONH_2_	917.4505	917.4537	90	23.6
S-3	Trp-Arg-(D-1-Nal)-Arg-Trp-CONH_2_	899.4787	899.4154	99	22.9
S-4	Trp-Arg-(F_5_-Phe propionic acid)-Arg-Trp-CONH_2_	939.4160	939.4189	98	24.4
S-5	Trp-Arg-(8-AOA)-Arg-Trp-CONH_2_	843.5100	843.5135	91	21.4
S-6	Trp-Arg-(12-ADO)-Arg-Trp-CONH_2_	899.5726	899.5748	95	26.0
S-7	Arg-Trp-(12-ADO)-Trp-Arg-CONH_2_	899.5726	899.5743	96	24.6
S-8	Arg-(D-1-Nal)-(12-ADO)-(D-1-Nal)-Arg-CONH_2_	921.5821	921.5842	96	28.5
S-9	Arg-(D-1-Nal)-(8-AOA)-(D-1-Nal)-Arg-CONH_2_	865.5195	865.5238	94	25.4

^a^% Purity based on RP-HPLC area under the peak, ^b^retention time in RP-HPLC run.

Previous structure-activity studies have emphasized on the role of backbone residue arrangement to alter hydrophobicity and consequently the antibacterial activity/selectivity of peptidomimetics^40^. Therefore, we rearranged the template to Arg-X-U_n_-X-Arg (where, U_n_ = 12-ADO and X = Trp (S-7) or D-1-Nal (S-8)) giving rise to bolaamphiphilic arrangement with a hydrophobic core and cationic Arg residues placed at both ends of the molecule. Compared to linear amphiphiles which carry a charge at one end of the molecule, bolaamphiphiles exhibit decreased insertion into the bulk of lipid bilayers^22,41^ which improve cell selectivity of such molecules. Corroborating this it has been reported that bolaamphiphilic arrangement mitigates toxicity and improves cell penetration for several antibacterial as well as cell penetrating peptides/peptidomimetics^22,42^. Encouragingly cationic bolaamphiphilic molecules such as brilacidin and XF-73 which primarily operate through membrane disruption mechanism are currently under clinical trials for Gram-positive infections^22^. Hence, as expected, compared to S-6 the bolaamphiphilic arrangement of same residues led to lower hydrophobicity (retention time) for S-7 (Table 1). While the combination of two D-1-Nal residues and 12-ADO imparted S-8 with the highest retention time and thus hydrophobicity among the designed peptidomimetics (Table 1). In S-9, the central 12-ADO moiety was replaced with shorter chain 8-AOA while keeping D-1-Nal and Arg residues intact. S-9 showed relatively lower retention time as compared to S-8. All the designed peptidomimetics were synthesized using solid phase peptide synthesis strategy (Fig. 1). Table 1 summarizes the name, mass, % purity and RP-HPLC retention time for designed peptidomimetics. The RP-HPLC chromatograms and mass spectral characterization data for all peptidomimetics are provided in electronic supplementary information (ESI) [ESI Fig. S1 and ESI Fig. S2].

### Antibacterial activity of designed peptidomimetics

The purified peptidomimetics were screened for antibacterial activity against a panel of staphylococcal strains. The results showed that S-1, S-2, S-3, and S-5 were inactive up to the maximum concentration tested of 45.4 μg/mL (Table 2). S-4 showed modest activity against the tested staphylococcal strains with minimum inhibitory concentration (MIC) at 45.4 μg/mL. Compared to template sequence S-1, S-6 and S-7 showed good activity against all the tested staphylococcal strains with MIC at 11.3 μg/mL, except *S. aureus* ATCC 25923 against which the MIC for both peptidomimetics was found to be 22.7 μg/mL. S-8 showed excellent activity against the tested strains with MIC in the concentration range of 0.3–1.4 μg/mL. To dissect whether the improved potency of S-8 was due to the incorporation of 12-ADO moiety or D-1-Nal residues, we designed S-9 with 8-AOA as U_n_ moiety. The modest activity of S-9 (MIC: 22.7- ≥ 45.4 μg/mL) confirmed a major role of 12-ADO moiety in imparting overall potency to the peptidomimetics S-6, S-7 and S-8. The standard antibiotics oxacillin (OXA) and vancomycin (VAN) were also assayed under identical conditions. OXA exhibited excellent potency against methicillin-sensitive *S. aureus* strains but was less active against MRSA while VAN showed potent activity against all the tested staphylococcal strains (Table 2).

**Table 2:** Minimum inhibitory concentration (MIC) of designed peptidomimetics against staphylococcal strains.

Name	Minimum inhibitory concentration, MIC (μg/mL)						
	*S. aureus* (ATCC 29213)	*S. aureus* (ATCC 25923)	*S. aureus* (ISP479C)	*S. aureus* (ISP479R)	MRSA (ATCC 33591)	MRSA(ATCC 43300)	MRSE^a^ (ATCC 35984)
S-1	>45.4	>45.4	ND	ND	>45.4	>45.4	>45.4
S-2	>45.4	>45.4	ND	ND	>45.4	ND	ND
S-3	>45.4	>45.4	ND	ND	>45.4	ND	ND
S-4	45.4	45.4	45.4	45.4	45.4	ND	ND
S-5	>45.4	>45.4	ND	ND	>45.4	ND	ND
S-6	11.3	22.7	11.3	11.3	11.3	11.3	11.3
S-7	11.3	22.7	11.3	11.3	11.3	11.3	11.3
S-8	0.7	1.4	0.3	0.7	1.4	1.4	1.4
S-9	22.7	22.7	45.4	45.4	22.7	>45.4	45.4
VAN	0.7	1.4	0.3	0.3	1.4	0.7	2.8
OXA	< 0.3	< 0.3	0.3	0.3	56.8	56.8	>45.4

^a^Methicillin-resistant *S. epidermidis*; ND, not determined; VAN, vancomycin; OXA, oxacillin.

Vancomycin-resistant enterococci (VRE) are another class of clinically relevant human pathogens^43^. For the active peptidomimetics (S-6, S-7 and S-8) we further evaluated their efficacy against four *Enterococcus* strains (Table 3). The data revealed good activity of peptidomimetics S-6 (MIC: 11.3–22.7 μg/mL) and S-7 (MIC: 5.6–22.7 μg/mL) while S-8 again showed most potent efficacy with MIC in the concentration range of 1.4–5.6 μg/mL (Table 3). Of note, both the clinical antibiotics VAN (MIC: >45.4 μg/mL) and OXA (MIC: >45.4–232.7 μg/mL) were ineffective against the tested VRE strains.

**Table 3:** Minimum inhibitory concentration (MIC) of active peptidomimetics against enterococcal strains.

Name	Minimum inhibitory concentration, MIC (μg/mL)			
	*E. faecalis* (ATCC 29212)	*E. faecium*^a^ (ATCC 700221)	*E. faecium*^b^ (E-447)	*E. faecium*^b^ (E-7846)
S-6	22.7	22.7	11.3	11.3
S-7	11.3	22.7	11.3	5.6
S-8	2.8–5.6	2.8	1.4–2.8	5.6
VAN	1.4	>45.4	>45.4	>45.4
OXA	5.6	>45.4	>232.7	>232.7

^a^Vancomycin-resistant *E. faecium*, ^b^clinical isolates of vancomycin-resistant *E. faecium*.

Further, we tested the efficacy of designed peptidomimetics against a representative Gram-negative bacterial strain *Escherichia coli* (ATCC 11775). Encouragingly, S-8 exhibited growth inhibitory potential against *E. coli* with MIC at 5.6 μg/mL while S-6 and S-7 were found to be inactive up to the maximum concentration tested of 45.4 μg/mL (data not shown here).

For many clinically used antibiotics, initial inoculum dependent efficacy^44^ has been reported while the presence of complex blood matrix (serum/plasma) is known to hamper activity of most of the naturally occurring linear CAMPs owing to proteolysis or through binding with serum proteins^45^. Therefore, we evaluated the efficacy of active peptidomimetics against higher inoculums and after preincubation with mice plasma and fetal bovine serum (FBS). Upon using 10^7^ CFU/mL as initial inoculum, no change in MIC was observed for S-6 and S-7 while for S-8 and VAN a two-fold increase in MIC was observed (Table 4). After 3 h pre-incubation with serum/plasma (50% v/v) both S-6 and S-8 showed a maximum two-fold increase in MIC (Table 4). For S-7 there was a two-fold increase in MIC upon incubation with serum while in presence of mice plasma activity was further reduced as the observed MIC was found to be >45.4 μg/mL (Table 4). Thus, with a completely unnatural backbone, S-8 maintained robust efficacy to withstand proteolysis and avoid serum/plasma inactivation under the experimental conditions tested (Table 4). The comparator antibiotic VAN also maintained its MIC in presence of serum/plasma.

**Table 4:** Minimum inhibitory concentration (MIC) of active peptidomimetics against MRSA ATCC 33591 under various incubation conditions.

Name	Minimum inhibitory concentration, MIC (μg/mL)		
	10^7^ CFU/mL	Fetal bovine serum (50%, v/v)	Mice plasma (50%, v/v)
S-6	11.3	22.7	22.7
S-7	11.3	22.7	>45.4
S-8	2.8	1.4–2.8	2.8
VAN	2.8	1.4	1.4

### The designed peptidomimetics were non-hemolytic and non-toxic towards mammalian cells

**Figure 2:**
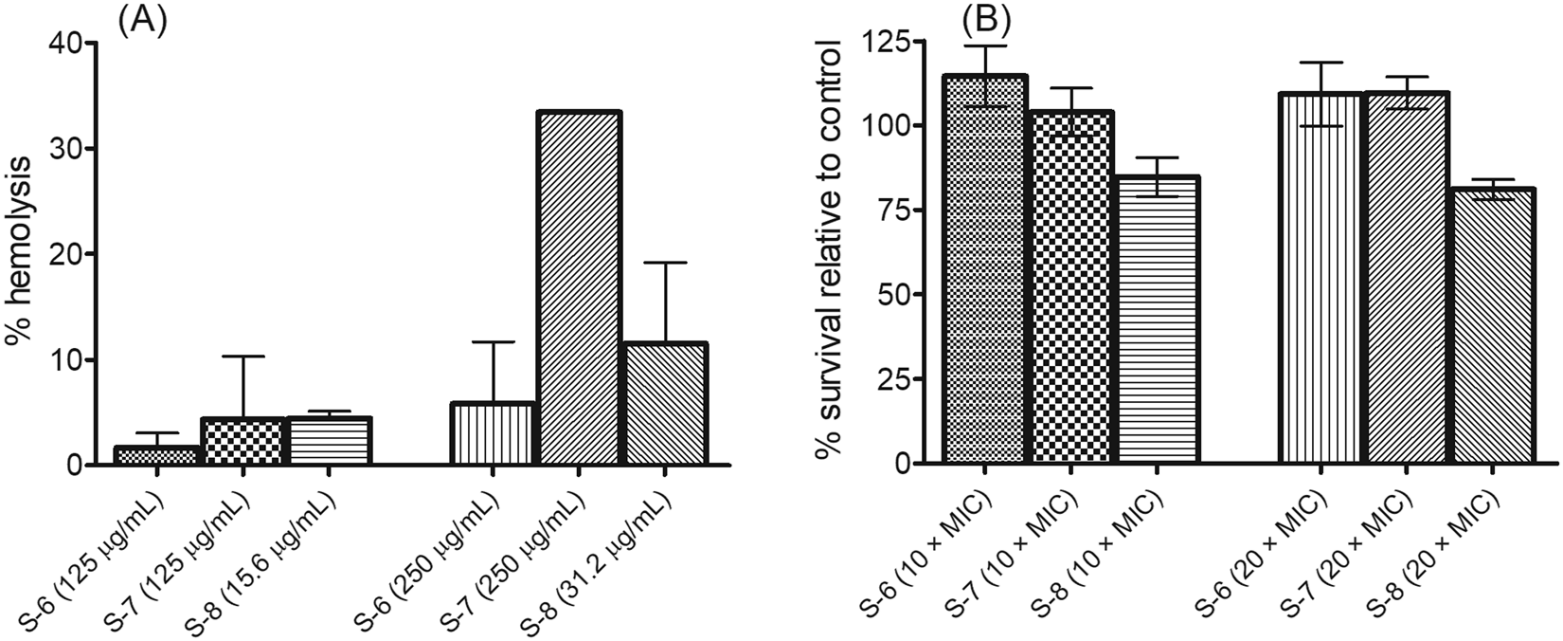
Hemolysis and cytotoxicity assay. (**A**) The percentage hemolysis of mice RBC upon treatment with active peptidomimetics. For the experiment, 0.1% Triton X-100 was used as positive control. The experiments were performed in duplicate on two different days and each data point represents mean ± S.D. (**B**) Cytotoxicity induced upon incubation of active peptidomimetics with 3T3 murine fibroblast cell line. The percentage viability of cells relative to untreated growth control was measured using MTT assay. The experiments were performed in triplicate on two different days and each data point represents mean ± S.D. The MIC values for test peptidomimetics against MRSA ATCC 33591 were as follows: S-6 (11.3 μg/mL), S-7 (11.3 μg/mL) and S-8 (1.4 μg/mL).

For evaluation of cell specificity, we measured % hemolysis caused by designed peptidomimetics on mouse red blood cells (RBCs). The results showed that S-1, S-2, S-3, S-4, S-5 and S-9 caused <5% hemolysis up to 250 μg/mL (data not shown here). For active peptidomimetics S-6, S-7 and S-8 we measured % hemolysis at concentrations slightly higher than 10× and 20× their respective MICs against MRSA ATCC 33591 strain. The results showed that all three peptidomimetics caused <10% hemolysis at concentrations ~10× MIC. At concentrations ~20× MIC, the % hemolysis was found to be 5.8 ± 5.8% for S-6 (at 250 μg/mL), 33.4 ± 0.0% for S-7 (at 250 μg/mL) and 11.5 ± 7.6% for S-8 (at 31.2 μg/mL) (Fig. 2A). Further, results of cytotoxicity assay showed that treatment with S-6 and S-7 at 10× and 20 × MIC resulted in no detrimental effects as no decrease in cell viability was observed relative to untreated control (Fig. 2B). S-8 treatments at 10× and 20 × MIC resulted in 84.8 ± 5.6% and 81.1 ± 2.9% viable cells, respectively. Thus, the designed peptidomimetics showed little to no toxicity towards mammalian cells at concentrations much higher than MIC except S-7 which caused 33.4% hemolysis at 250 μg/mL.

In various structure-activity relationships on CAMP mimics a direct influence of increased hydrophobicity on improvement in antibacterial efficacy has been reported^30,36^. However, in the present work as only subtle changes in hydrophobicity were observed among designed peptidomimetics, the structure-activity relationship revealed no direct associations (Tables 1–3). Although as expected, the most hydrophobic peptidomimetic S-8 showed most potent and broad-spectrum efficacy. Of note, S-7 and S-8 were based on the same template and carried same net charge (+3) yet S-8 showed better potency which may be due to the nature of hydrophobic moieties i.e., Trp in S-7 and D-1-Nal in S-8. Previous studies on CAMP mimics have also shown that increasing aromatic ring size improves potency as replacement of Trp residue with slightly larger D-1-Nal moiety has been shown to improve efficacy^27^ while incorporation of Tyr residue in place of Trp was found to be detrimental to antibacterial potency^46^. Further, since S-8 contained both L- and D-residues the configurational effects might as well be contributing towards its activity/cell selectivity as it has previously been reported that compared to all L-amino acid containing peptidomimetics corresponding mixed D/L- analogs show improved cell selectivity^47^.

### Mechanism of action against exponential phase MRSA ATCC 33591 strain

**Figure 3:**
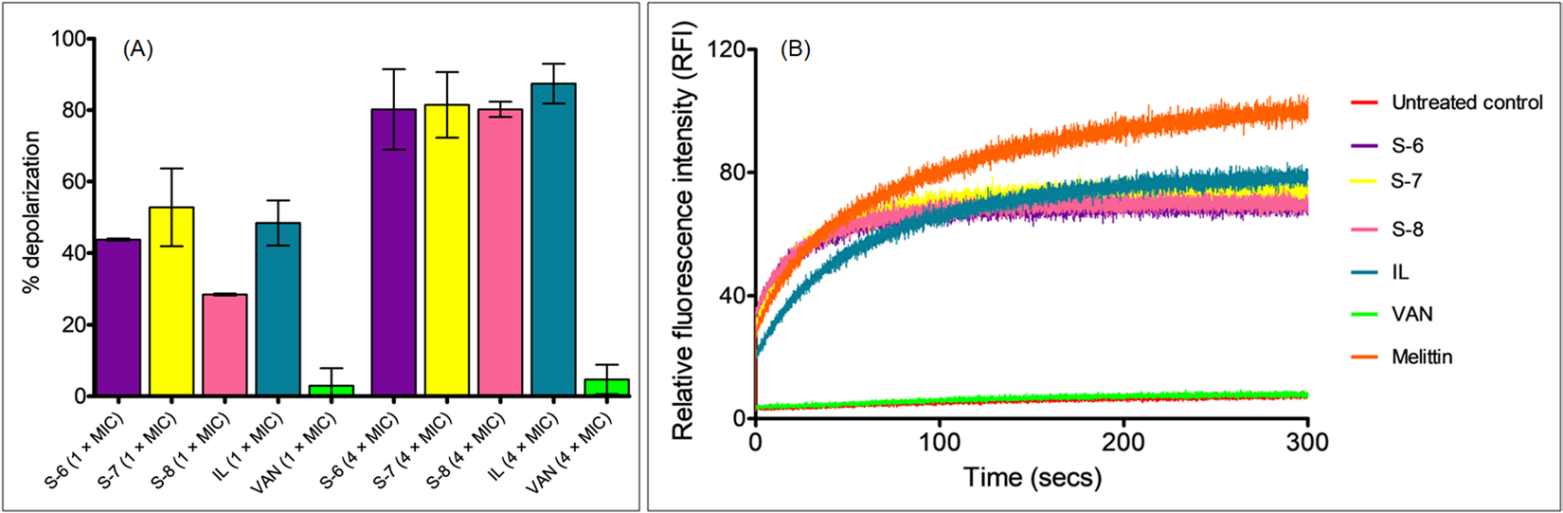
Membrane depolarization mechanism of designed peptidomimetics against MRSA ATCC 33591 strain assessed using membrane potential sensitive dye DiSC_3_(5). (**A**) Percentage depolarization caused by treatment of dye-loaded cells with different concentrations of test agents after 2 min incubation. The % depolarization was calculated relative to melittin (10 μM) as positive control. The experiments were repeated on two different days and mean ± S.D. is presented here. (**B**) Depolarization kinetics monitored at 4 × MIC concentration of test agents. The experiment was repeated twice and similar data was obtained. Representative data from one set is shown here. The MIC values for various test agents against MRSA ATCC 33591 strain were as follows: S-6 (11.3 μg/mL), S-7 (11.3 μg/mL), S-8 (1.4 μg/mL), IL (3.2 μg/mL) and VAN (1.4 μg/mL).

Towards elucidation of mechanism we evaluated the effect of designed peptidomimetics on membrane potential in MRSA using a membrane potential sensitive dye 3,3’- dipropylthiadicarbocyanine iodide (DiSC_3_(5)) as a probe^48^. The results showed that relative to melittin control (set as 100%), at MIC, the % depolarization was found to be 43.7 ± 0.3% for S-6, 52.7 ± 10.8% for S-7, 28.4 ± 0.2% for S-8, 48.3 ± 6.3% for indolicidin (IL, MIC: 3.2 µg/mL against tested MRSA strain) and 2.9 ± 4.8% for VAN treatment (Fig. 3A). At 4 × MIC, the % depolarization increased to 80.1 ± 11.2% for S-6, 81.4 ± 9.1% for S-7, 80.2 ± 2.1% for S-8 and 87.4 ± 5.5% for IL while for VAN treatment there was minimal increase with 4.7 ± 4.1% depolarization (Fig. 3A). The depolarization trend observed for melittin, IL and VAN is in line with previous literature as it has been reported that melittin and IL cause immediate dissipation while VAN does not interfere with membrane potential in *S. aureus*^12,49,50^. We further monitored kinetics of depolarization at 4 × MIC to evaluate if there is time dependence for increase in relative fluorescence intensity (RFI) upon peptidomimetic treatment. The data showed an immediate increase (within ~2 min) in fluorescence intensity upon treatment for all test agents except VAN which showed RFI values similar to untreated control cells (Fig. 3B). Although the designed peptidomimetics showed comparable increase in RFI the relative order of increase was S-7 > S-8 > S-6 (Fig. 3B). Representative emission spectrum for DiSC_3_(5) probe standardization in different environments is given in ESI Fig. S3.

In parallel, in viability assay with dye-loaded cells in HEPES-glucose buffer at 4 × MIC, all treatments caused significant reductions (>3 log reduction, p < 0.05) in cell count except VAN which caused 0.6 log reduction in viability relative to untreated control (ESI Fig. S4). Thus, a direct correlation was observed between depolarization kinetics and viability for active peptidomimetics under the experimental conditions.

**Figure 4:**
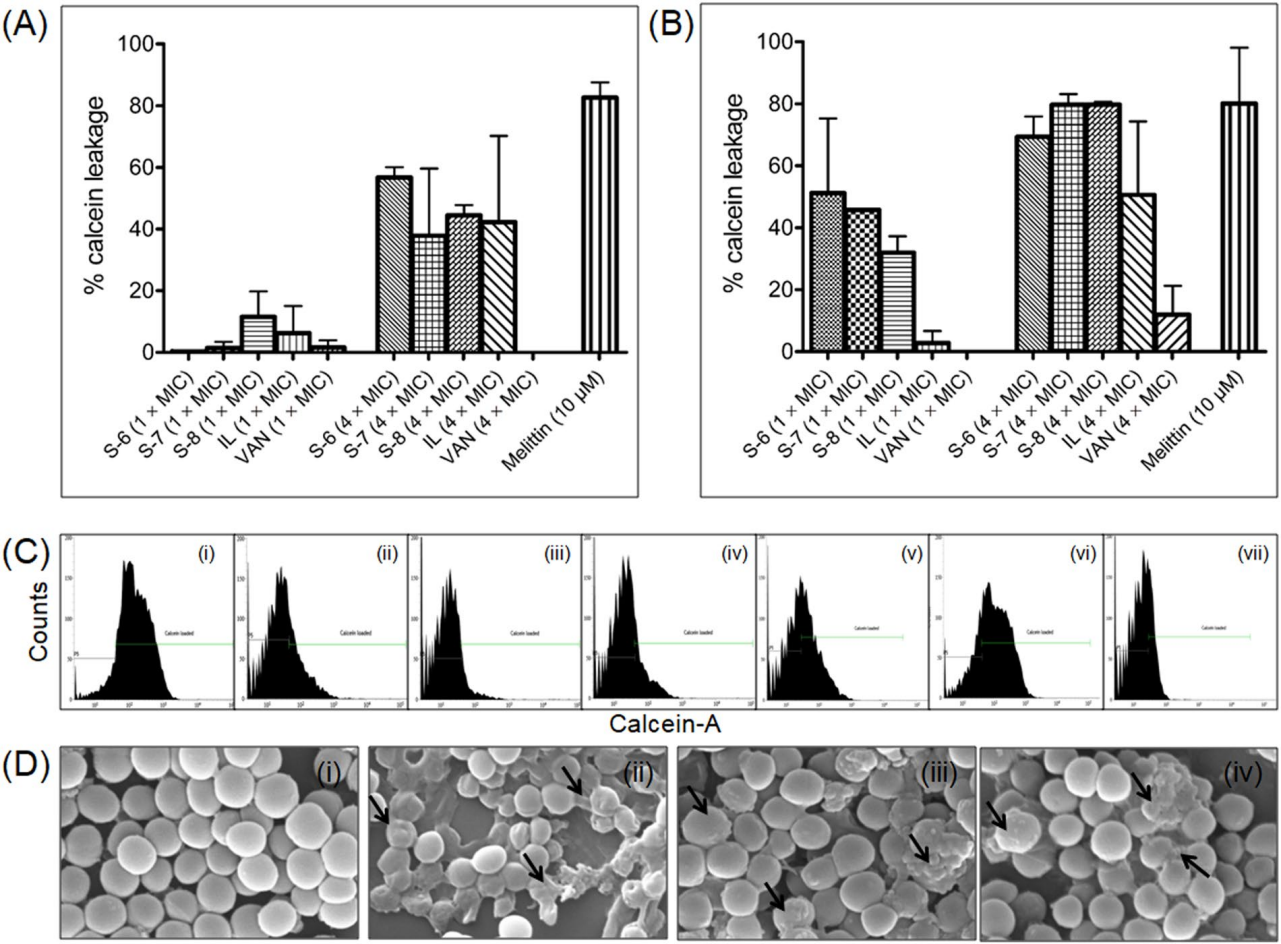
Membrane permeabilization mechanism of designed peptidomimetics against MRSA ATCC 33591 strain. Percentage calcein release caused by treatment of dye-loaded cells with different concentrations of test agents upon 2 min incubation (**A**) and 2 h incubation (**B**). The percentage leakage was calculated relative to untreated control loaded cells. The experiments were repeated on two different days and mean ± S.D. is presented here. (**C**) Representative histograms of calcein-loaded cells incubated with different test agents at 4 × MIC for 2 h. (i) Untreated control, (ii) S-6 (45.4 μg/mL), (iii) S-7 (45.4 μg/mL), (iv) S-8 (5.6 μg/mL), (v) IL (12.8 μg/mL), (vi) VAN (5.6 μg/mL) and (vii) melittin (10 μM). A total of 10,000 cells were acquired for each flow cytometry analysis. (**D**) Scanning electron microscopy images of ultrastructural effects observed on MRSA ATCC 33591 strain after incubation with designed peptidomimetics. The cells were incubated with different peptidomimetics for 4 h at respective 10 × MIC concentrations. (i) Untreated control, (ii) S-6 (113 μg/mL), (iii) S-7 (113 μg/mL), and (iv) S-8 (14 μg/mL). The arrows indicate morphological alterations caused by treatment. The experiment was repeated on two different days and similar effects were observed. Representative data is presented here.

We next assessed membrane permeabilization efficacy using non-fluorescent calcein-AM dye as a probe^51^. The results showed that at MIC, upon 2 min incubation, all test agents caused minimal permeabilization as the % leakage observed was 0.28 ± 0.39% for S-6, 1.4 ± 1.9% for S-7, 11.5 ± 8.2% for S-8, 6.2 ± 8.8% for IL and 1.6 ± 2.3% for VAN (Fig. 4A). At 4 × MIC, upon 2 min incubation the % leakage increased to 56.7 ± 3.3% for S-6, 37.8 ± 21.7% for S-7, 44.4 ± 3.2% for S-8 and 42.2 ± 27.9% for IL, while, VAN did not cause any leakage at 4 × MIC (Fig. 4A). In comparison, melittin treatment (10 μM) immediately caused 82.6 ± 4.9% dye leakage upon 2 min incubation. We further evaluated the effect of incubation time on dye release and the results showed that at MIC upon 2 h incubation, the % dye leakage was found to be 51.1 ± 24.1% for S-6, 45.7 ± 0.0% for S-7, 31.9 ± 5.2% for S-8 and 2.7 ± 3.9% for IL. At 4 × MIC, upon 2 h incubation the permeabilization effect increased further as the % dye leakage was observed to be 69.3 ± 6.5% for S-6, 79.6 ± 3.4% for S-7, 79.7 ± 0.9% for S-8, 50.6 ± 23.6% for IL and 11.9 ± 9.2% for VAN (Fig. 4B). For melittin (10 μM) upon increasing the incubation time to 2 h the % leakage was found to be 80.1 ± 17.9%. Thus, in line with previous literature melittin caused immediate membrane leakage while IL exhibited concentration-dependent increase in dye leakage^49,50^. In comparison, the designed peptidomimetics showed both concentration as well as time-dependent increase in dye leakage. Figure 4C depicts representative histograms of calcein-loaded cells after treatment with test agents at 4 × MIC upon 2 h incubation. As can be seen from the histograms, after treatment there is a clear shift in dye fluorescence from higher to lower values indicative of dye release except for VAN treatment which did not elicit membrane permeabilization (Fig. 4C). The viable cell counts corresponding to calcein leakage assay at 4 × MIC also showed more than 2 log reduction in viability after 2 h incubation, confirming leakage to be a lethal event for the designed peptidomimetics (ESI Fig. S5). Under identical conditions IL caused 1.8 log reductions, VAN caused no reduction and melittin caused complete eradication of cells (ESI Fig. S5).

To further assess if membrane depolarization and permeabilization caused by active peptidomimetics may induce surface perturbations we visualized alterations in surface integrity of MRSA using scanning electron microscopy (SEM). The results showed that the untreated control cells exhibit bright and smooth surface with round morphology (Fig. 4C(i)). After treatment with all three peptidomimetics for 4 h, massive losses in surface integrity resulting from pore formation ultimately leading to flattening of cells was visible (Fig. 4C(ii-iv)).

Additionally, to explore the possibility that cationic nature of the designed peptidomimetics might allow them to interact with other negatively charged intracellular components such as DNA, we performed comparative DNA gel retardation experiment for S-7 and S-8. The results showed that S-7 was able to retard DNA movement near its MIC (12.5 μg/mL) while S-8 required ~7 × MIC concentration (10 μg/mL) to cause retardation in DNA movement (ESI Fig. S6).

Thus, overall the mechanistic work involving membrane depolarization and calcein leakage experiments showed that at MIC upon 2 min incubation all three active peptidomimetics were able to traverse the peptidoglycan layer and reach the cytoplasmic membrane causing transient dissipation in membrane potential although membrane integrity was maintained as negligible calcein leakage was observed. Upon increasing concentration to 4 × MIC, all three peptidomimetics caused immediate dissipation of membrane potential (~80%) within 2 min while the extent of permeabilization also increased (<60%), although almost 2 h incubation was required to cause ~70–80% leakage. Thus, immediate membrane depolarization followed by permeabilization was observed. The time lag between membrane depolarization and permeabilization may be due to the fact that calcein being a relatively large probe requires pores of finite size in bacterial membranes to escape, while, dissipation of membrane potential may lead to transient alterations in membrane permeability causing leakage of small solutes such as ions and ATP only^23,50^. The membrane perturbation mechanism for designed peptidomimetics was further corroborated by electron microscopy observations as severe breaches in surface morphology, leakage of cellular contents and flattening of cells were observed after treatment. Thus, the mechanistic studies established that similar to membrane active CAMPs^16,52^ the primary mode of action for designed active peptidomimetics is membrane depolarization followed by permeabilization ultimately leading to cell disruption/lysis and loss of viability. Further, at present, based on DNA retardation experiment intracellular targeting cannot be ruled out for S-7.

**Figure 5:**
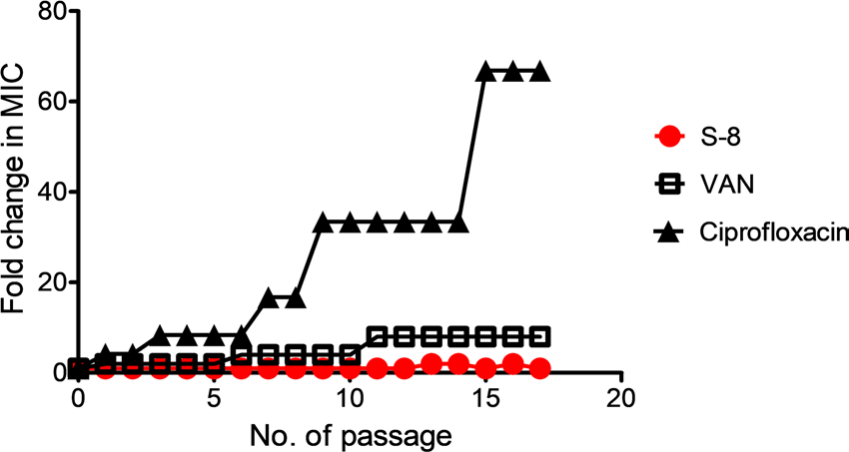
*In vitro* resistance development study against MRSA ATCC 33591 strain. Changes in MIC upon 17 serial passages with sub-MIC concentrations of S-8, VAN and ciprofloxacin were monitored. The fold change in MIC represents the ratio of the MIC after each passage to the initial MIC before the first passage.

### MRSA did not develop resistance against S-8 over 17 serial passages

Recently growing number of reports are demonstrating that multiple resistance mechanisms are rendering CAMPs ineffective^19^. Therefore, for S-8 which showed maximum efficacy against all the tested bacterial strains, we evaluated propensity of MRSA ATCC 33591 strain to develop resistance against S-8 over 17 serial passages at sub-MIC concentrations. Ciprofloxacin (CIP) belongs to fluoroquinolone class of antibiotics which has been shown to develop resistance rapidly under *in vitro* conditions^24,29,53^. Therefore, for the experiment we used CIP as positive control. Upon serial passage for S-8 the data showed a maximum 2-fold increase in MIC at passage 13, however, the increase was not stable up to the 17^th^ passage (Fig. 5). As a control, we also tested resistance development propensity for VAN under identical conditions. For VAN an 8-fold increase in MIC was observed at the 11^th^ passage which remained constant up to the 17^th^ passage. For CIP, a rapid increase in MIC was observed with almost 16-fold increase in MIC at 6^th^ passage. The initial MIC of CIP was 0.17 μg/mL and after 17 passages, the final MIC observed was 11.3 μg/mL resulting in 66.8 fold increase in MIC over the duration of the experiment. Interestingly, for the CIP-treated MRSA strain starting from passage 3, the cell growth was significantly slower and the colony morphology was also relatively smaller as compared to the untreated control bacterial line (data not shown here). Thus, while the tested MRSA strain showed lesser propensity to develop resistance against S-8 the clinical antibiotic CIP rapidly selected resistant mutants under tested conditions. The results obtained with CIP and VAN are in line with previously published reports for resistance development on different *S. aureus* strains^53,54^.

### Effect of bacterial growth phase and nutrient medium on bactericidal efficacy

**Figure 6:**
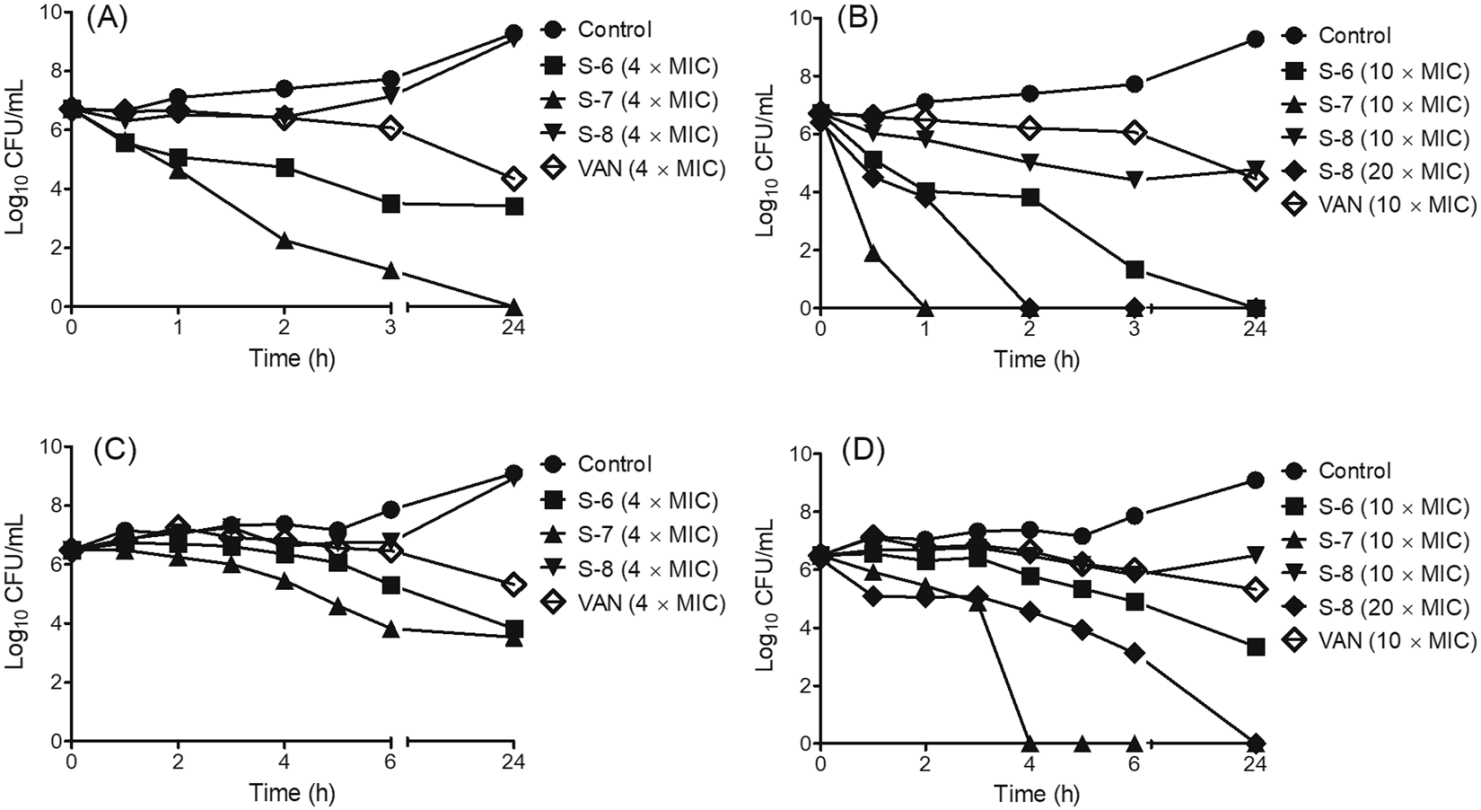
Bacterial killing kinetics of designed peptidomimetics against exponential and stationary phase MRSA ATCC 33591 in MHB or nutrient-depleted MHB medium. The active peptidomimetics were incubated with exponential phase cells in MHB medium (**A** and **B**) or stationary phase cells in nutrient depleted medium (**C** and **D**) at 37 °C with shaking. Appropriate controls without treatment were also run in parallel. At desired time point a small aliquot was removed and diluted in PBS before plating on BHI agar plates. The number of viable CFU was enumerated following 16–18 h incubation of plates at 37 °C. The experiments were repeated on two different days and similar data was obtained. Representative data is shown here.

After establishing activity and mechanism against planktonic cells we next evaluated the efficacy of designed peptidomimetics against MRSA biofilms. In biofilm mode of growth due to nutrient-depletion and growth phase variations the physiology of bacterial cells gets altered^55^, therefore, we first determined the potency of designed peptidomimetics against exponential phase cells in normal medium and stationary phase cells in nutrient-depleted MHB medium. The results of time-dependent killing against exponential phase MRSA at 4 × MIC showed that S-6 exhibited bactericidal effect (>3 log reduction in viability) upon 3 h incubation, while S-7 achieved 4.4 log reduction in viability within 2 h incubation (Fig. 6A). For S-8 treated cells, at 4 × MIC, a minimal decrease was observed in cell viability up to 2 h and after 2 h re-growth occurred leading to cell counts almost equal to the untreated control at 24 h (Fig. 6A). A similar re-growth effect has been previously reported for various antibiotics/synthetic lead molecules at low multiples of MIC^56,57^. At 10 × MIC, S-6 was able to cause 5.3 log reductions in viable counts upon 3 h incubation while S-7 caused complete eradication within 1 h (Fig. 6B). At 10 × MIC, S-8 caused 1.9 log reductions upon 24 h incubation (Fig. 6B). To assess the effect of increasing concentration, we further performed the kill kinetics experiment for S-8 at 20 × MIC, at which it achieved rapid bactericidal end point with complete eradication of cells within 2 h incubation (Fig. 6B). For S-8, the higher concentration verge required to show bactericidal effect in growth medium (20 × MIC) could partially be due to the higher initial inoculum used for bactericidal killing experiments as a two-fold increase in MIC value was observed for S-8 against 10^7^ CFU/mL initial inoculum (Table 4). In this context, it is also pertinent to consider that compared to S-6 and S-7, the MIC of S-8 is ~8-fold lower and thus to have a conventional membrane disruptive carpet mechanism^16^ relatively higher concentration threshold is obligatory for this peptidomimetic to achieve bactericidal end point. Under the experimental conditions, the standard antibiotic VAN also caused ~2.3 log reduction in CFU/mL at 4 × and 10 × MIC concentrations over 24 h.

Further, against stationary phase cells in nutrient-depleted medium a relatively slower rate of killing was observed as at 4 × MIC, S-6 caused only 2.6 log reduction while S-7 also caused 2.9 log reductions in CFU/mL after 24 h incubation (Fig. 6C). At 4 × MIC, S-8 showed no growth inhibition up to 6 h while at 24 h time point again re-growth was observed. At 10 × MIC, S-6 caused a gradual decrease in the number of viable cells with 3.1 log reduction after 24 h incubation while S-7 treatment led to a biphasic kill curve with initial slow killing up to 3 h (Fig. 6D). However, upon 4 h incubation, bactericidal effects were observed for S-7 with complete eradication of cells. Relative to initial inoculum for S-8 at 10 × MIC no reduction in the viable count was observed up to 6 h incubation (Fig. 6D). However, at 20 × MIC, S-8 treatment also led to complete eradication of cells upon 24 h incubation (Fig. 6D). Under the experimental conditions VAN caused ~1.1 log reduction in CFU/mL after 24 h incubation at both 4× and 10 × MIC. Thus, no concentration dependence was observed for VAN under nutrient-depleted conditions. The poor efficacy of VAN against stationary phase cells in nutrient-depleted media is in line with previously reported literature^12,13^. Further, a drop in killing efficacy under nutrient-depleted conditions has previously been reported for membrane-active dual targeting antibiotics oritavancin and daptomycin as well^12,13^, while even exclusively membrane targeting synthetic β-peptoid peptide hybrid oligomers have also been reported to exhibit a reduced rate of killing under similar conditions^58^. As reported for antibiotics, the decreased rate of killing might originate from presence of tolerant persister cells predominating under nutrient-depleted stationary phase conditions^5,55^. Alternatively, the nutrient-depleted medium might lead to the accumulation of components which hamper killing by designed peptidomimetics, although further investigations are needed to draw a conclusion.

It is of interest to note here that the designed peptidomimetics caused dissipation of membrane potential within 2 min of incubation while in killing kinetics in growth medium the rate of killing was relatively slower (Figs 3B and 6A). This may be due to the ongoing replication occurring during the course of the incubation of peptidomimetics with MRSA in the killing kinetics experiment. Similar to our data it has previously been reported that compared to a buffer, in nutrient-rich growth medium, bacteria can repair peptide-mediated membrane damage and recover growth more easily^59^.

Based on bactericidal kinetics data the effect of the alternate and bolaamphiphilic template was observed for S-6 and S-7 as although both comprise of identical residues and exhibit same MIC against MRSA, yet mechanistically S-7 showed more rapid bactericidal kinetics which might be attributed to its slightly better membrane permeabilization efficacy (Fig. 4B). As S-6 did not cause complete eradication of stationary phase cells over 24 h, for further work against MRSA biofilms we studied the efficacy of S-7 and S-8 only.

### Efficacy of designed peptidomimetics against 24 h and 48 h mature MRSA biofilms

**Figure 7:**
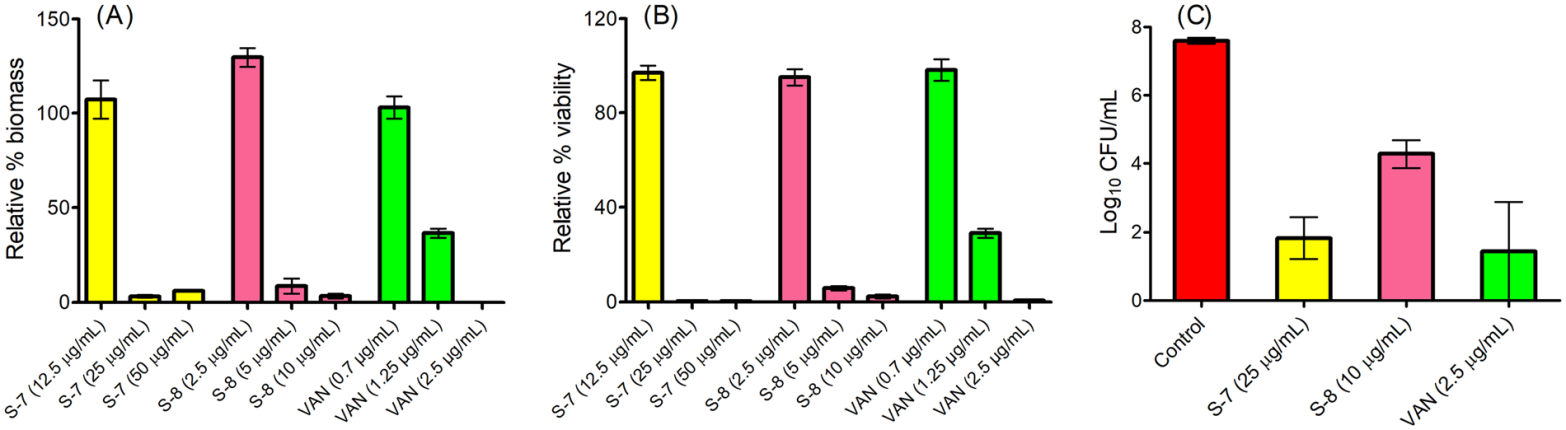
Biofilm formation inhibition assay for designed peptidomimetics against MRSA ATCC 33591 strain. In 96-well plates, biofilms were allowed to grow in absence or presence of different concentrations of active peptidomimetics and VAN. After 24 h the biofilms were washed once with PBS and subjected to different assays. (**A**) % biomass quantified using crystal violet staining assay and (**B**) percentage viability quantified using Alamar blue reagent fluorescence. (**C**) The number of viable cells was quantified using viable count assay. The experiment was repeated twice and each data point in panels (A) and (B) represents mean ± S.E. from twelve replicates. The viability data in panel (C) represents mean ± S.E. from two independent experiments performed on two different days. For panel C, statistical differences from control values were determined by one-way analysis of variance (ANOVA) with Bonferroni’s multiple-comparison post hoc tests. The differences between the control vs. S-7 and control vs. VAN were statistically significant (p < 0.05).

Along with slow growth under nutrient depletion conditions, biofilm-associated infections are complicated by the presence of extracellular polymeric substance (EPS) matrix which provides a physical barrier and allows bacterial populations to adhere together^7^. For a number of antibiotics and synthetic molecules, much higher concentrations have been reported to be effective towards eradication of biofilms^5,10,24,60,61^ while sub-lethal concentrations of many antibiotics such as VAN and OXA are even known to promote staphylococcal biofilm formation on surfaces^62^. To evaluate the efficacy of designed peptidomimetics we next developed a 24 h biofilm formation inhibition model in a 96-well plate. Results of crystal violet (CV) assay showed that at 12.5 μg/mL concentration S-7 was not effective towards biofilm eradication, however, at 25 μg/mL it effectively cleared the biomass (>95%) (Fig. 7A). S-8 treatment at 10 μg/mL and VAN treatment at 2.5 μg/mL also reduced biomass by >95% (Fig. 7A). Correspondingly, in alamar blue (AB) assay, S-7 treatment at 25 μg/mL led to 0.23% viable cells compared to untreated control (Fig. 7B). In AB assay S-8 treatment (10 μg/mL) led to 2.3 ± 0.7% viable cells while VAN treatment (2.5 μg/mL) was sufficient to reduce viability to <1% under identical conditions (Fig. 7B).

For the biofilm formation inhibition experiment at concentrations causing >95% reduction in biomass and viability we determined the corresponding viable counts. The number of cells in untreated control biofilm was found to be 7.59 ± 0.08 log CFU/mL (Fig. 7C). For treated samples, the remaining viable count was 1.82 ± 0.60 log CFU/mL for S-7 (25 μg/mL), 4.28 ± 0.40 log CFU/mL for S-8 (10 μg/mL) and 1.44 ± 1.44 log CFU/mL for VAN (2.5 μg/mL). Thus, S-7 at 25 μg/mL, S-8 at 10 μg/mL and VAN at 2.5 μg/mL effectively reduced biomass and viability (>95%) while showing bactericidal effects (>3 log reduction in viability compared to 24 h growth control).

**Figure 8:**
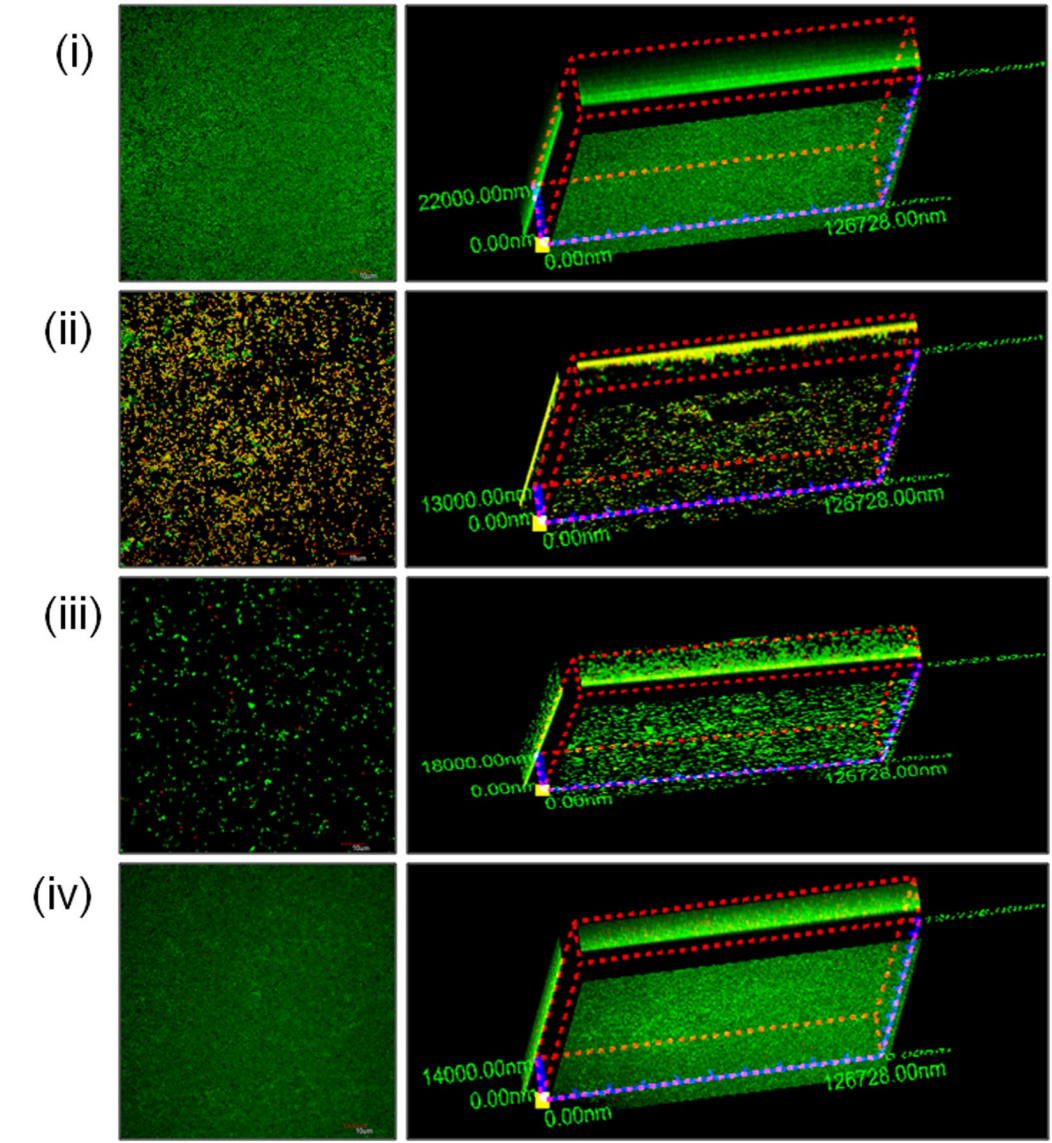
Efficacy of designed peptidomimetics and VAN against 48 h mature MRSA ATCC 33591 biofilms. Biofilms were grown in 8-well chamber slides for 24 h. After treatment (24 h) the biofilms were washed once with PBS and stained with Live/Dead viability assay kit [SYTO9 (green; viable cells) and propidium iodide (red; dead cells)] and were visualized using confocal laser scanning microscopy. (i) Untreated control, (ii) S-7 (20 × MIC, 226 μg/mL), (iii) S-8 (20 × MIC, 28 μg/mL) and (iv) VAN (20 × MIC, 28 μg/mL). The experiments were performed on three different days and similar data was obtained. Representative data is presented here.

We further measured the efficacy of designed peptidomimetics to eradicate preformed 48 h mature MRSA biofilms using confocal laser scanning microscopy. Towards this, we visualized the effect of active peptidomimetics on biofilms stained with Live/Dead viability assay kit which uses membrane permeability responsive DNA-binding dyes SYTO9 and propidium iodide (PI) as markers. Upon treatment, the green fluorescing bacterial population is indicative of live cells while the co-localization of green and red fluorescence indicates membrane permeabilized dead cells. For untreated control, the data in Fig. 8(i) demonstrated a dense lawn of green viable cells with a thickness of 22 μm (right panel). Upon treatment with S-7 at 20 × MIC (226 μg/mL) massive disruption in cell integrity was observed as permeabilized cells with co-localization of SYTO9 and PI fluorescence were visible (Fig. 8(ii)). Simultaneously, an appreciable decrease in biofilm thickness (13 μm) was also observed (Fig. 8(ii) right panel). Peptidomimetic S-8 treatment at 20 × MIC (28 μg/mL) also led to the disruption of biofilms as severe reduction in the number of cells was observed (Fig. 8(iii)). The 3-D image clearly showed that although the observed biofilm thickness was 18 μm, most of the biofilm matrix was devoid of cells (Fig. 8(iii) right panel). In comparison, VAN treatment at 20 × MIC (28 μg/mL) also led to a decrease in biofilm thickness (14 μm) although the entire thickness of biofilm maintained its robust architecture (Fig. 8(iv)).

**Figure 9:**
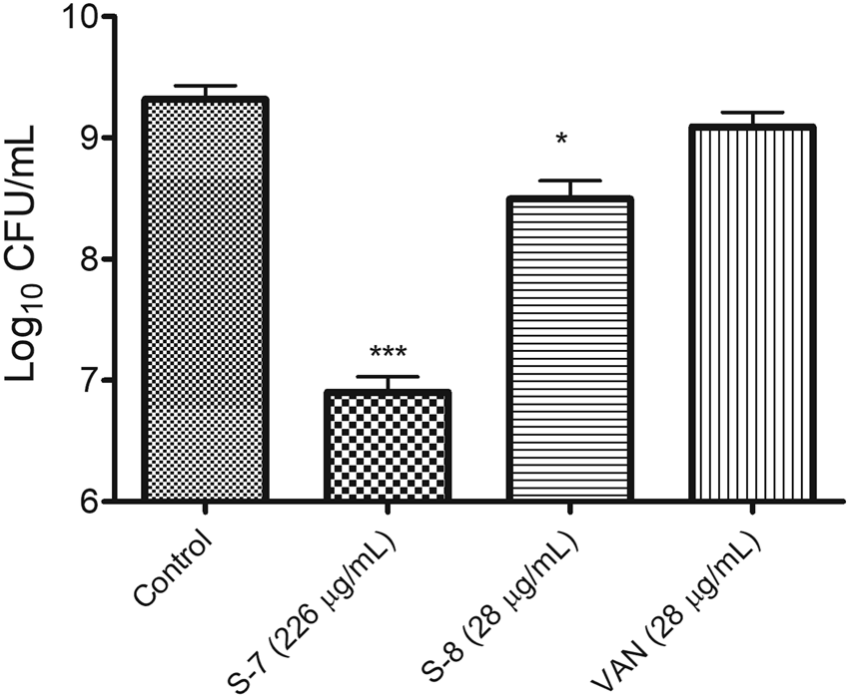
Cell viability corresponding to 48 h mature biofilm treatments. The viability of treated 48 h mature biofilm embedded MRSA cells relative to untreated control was quantified using drop plate method. The data represents mean ± S.D. from two independent experiments. Statistical differences from control values were determined by one-way analysis of variance (ANOVA) with Bonferroni’s multiple-comparison post hoc tests. The differences between the control vs. S-7 and control vs. S-8 were statistically significant (p < 0.05).

For quantitative analysis, corresponding to confocal microscopy experiment, we also enumerated the number of viable cells remaining after treatment with different peptidomimetics (at 20 × MIC). The results showed that untreated biofilms harbored 9.31 ± 0.11 log CFU/mL (Fig. 9). Upon treatment, a statistically significant decrease in the number of viable counts relative to untreated control was observed as the number of surviving cells was 6.90 ± 0.12 log CFU/mL for S-7 and 8.49 ± 0.15 log CFU/mL for S-8 (p < 0.05). Under the experimental conditions, VAN showed least efficacy with 9.08 ± 0.11 viable log CFU/mL upon treatment.

## Conclusion

In summary, using the sequence-template approach (5-mer, +3 charge, 60% hydrophobic residues, C-terminal amide) we developed 8 structurally novel peptidomimetics out of which, three peptidomimetics S-6, S-7 and S-8 containing 12-amino dodecanoic acid moiety were found to exhibit good to potent growth inhibitory activities against clinically relevant Gram-positive pathogens including MRSA, methicillin-resistant *S. epidermidis* and VRE. Encouragingly, the active peptidomimetics were cell selective and exhibited a primary bactericidal, membrane depolarizing/disrupting mechanism of action against MRSA. In present work, we used VAN as a comparator antibiotic which despite its increasing MIC values against MRSA is still considered a last resort drug in clinical settings. Our results showed that while the designed peptidomimetics S-7 and S-8 eradicated stationary phase MRSA cells in nutrient-depleted growth medium (>6 log reduction) and biofilms, VAN was least active under identical conditions (~1.1 log reduction and no disruption of biofilms). The data thus reinforced the findings that the designed membrane disruptive agents are better able to combat biofilms as compared to glycopeptide antibiotic VAN which has a single cellular target. Overall, the current study aimed at the development of a prospective class of anti-biofilm agents successfully delivers S-8 with consolidated potency against clinically relevant Gram-positive pathogens including VRE while also showing efficacy against representative Gram-negative bacteria *E. coli*. We envisage that, with low MIC, cell selectivity, proteolytic stability, membrane disruptive mode of action and low resistance development potential, S-8 warrants further structure-activity based optimization. At present, we are also investigating combinations of S-8 with different biofilm targeting quorum sensing agents and antibiotics to formulate a potential catheter lock solution effective against MRSA biofilms.

## Materials and Methods

### Chemicals

Rink amide-4-methylbenzhydrylamine (MBHA) resin and fluorenyl methoxy carbonyl (Fmoc)-protected amino acids (Fmoc-Trp(Boc)-OH and Fmoc-Arg(pbf)-OH) were purchased from Novabiochem (Darmstadt, Germany). *N,N*-dimethylformamide (DMF), *N*-methylpyrrolidinone (NMP), piperidine, trifluoroacetic acid (TFA) and HPLC grade solvents were purchased from Merck (Germany). 1-hydroxybenzotrizole (HOBt), *N*,*N*-diisopropyl carbodiimide (DIPCDI), unnatural Fmoc-amino acids, triisopropyl silane (TIS), crystal violet (CV), glucose, 3,3′-dipropylthiadicarbocyanine iodide (DiSC_3_(5)), calcein-acetoxymethyl ester (calcein-AM), bovine serum albumin (BSA), 3-(4,5-dimethylthiazol-2-yl)-2,5-diphenyltetrazolium bromide (MTT), Dulbecco’s modified Eagle medium (DMEM), dimethylsulphoxide (DMSO), vancomycin (VAN), and melittin were purchased from Sigma-Aldrich (USA). The Fmoc derivative of 12-amino dodecanoic acid was prepared using literature protocol^63^ and was characterized using RP-HPLC and LC-ESI-MS. Cation-adjusted Mueller-Hinton Broth (MHB) and tryptic soy broth (TSB) were purchased from Difco (USA). Brain heart infusion (BHI) medium and agar powder were purchased from Himedia (India). Live/Dead BacLight viability assay kit and Alamar blue (AB) reagent were purchased from Invitrogen (Eugene, OR). pBluescript II SK(+) phagemid kit was purchased from Agilent Technologies. DMF was double distilled prior to use.

### Bacterial strains and culture conditions

In this study following bacterial strains were used: *S. aureu*s (ATCC 29213), *S. aureus* (ATCC 25923), *S. aureus* ISP479C, *S. aureus* ISP479R, MRSA (ATCC 33591), MRSA (ATCC 43300), methicillin-resistant *Staphylococcus epidermidis* (ATCC 35984), *Enterococcus faecalis* (ATCC 29212), VRE (ATCC 700221), *E. coli* (ATCC 11775) and two clinical isolates of VRE [(E-447) and (E-7846)]. The VRE clinical isolates were a kind gift from Dr. Benu Dhawan at All India Institute of Medical Sciences, New Delhi, India. All the strains were stored as 20% (v/v) glycerol stock at −70 °C until sub-cultured on BHI agar plates for use.

## Methodology

### Solid phase peptide synthesis and purification

The designed peptidomimetics were synthesized either manually or using automated peptide synthesizer (Advanced Chemtech, USA) following standard Fmoc-chemistry on Rink amide-MBHA resin^64^ (loading 0.59 mmol/g). Briefly, coupling reactions were performed by gently swirling 4 equivalents of DIPCDI, HOBt, and respective amino acids for 0.5–4 h at room temperature. For monitoring each coupling and deprotection step, Kaiser test^65^ was performed. Upon completion of synthesis, to detach peptidomimetics from resin, a cleavage mixture comprising of trifluoroacetic acid/H_2_O/triisopropyl silane and phenol (95:2:2:1) was used. Purification of the crude peptidomimetics was performed on semi-preparative RP-HPLC column with a linear gradient of 10 to 90% acetonitrile (0.1% TFA) and water (0.1% TFA) over 45 min. The peptidomimetics after purification were characterized by LC-ESI-MS (WATERS SYNAPT G2 system). For comparison, sequence S-1 was also custom synthesized from Sigma-Aldrich (USA). For the biological activity experiments the peptidomimetics were dissolved in DMSO (10 mg/mL) and were diluted in autoclaved water to 1 mg/mL working stock concentrations.

### Minimum inhibitory concentration (MIC) determination

The antibacterial activity was evaluated as MIC using slight modifications to a serial broth micro dilution method as reported previously^66,67^. Briefly, to each treatment well of the 96-well plate (Corning Incorporated, USA) 10 μL of peptidomimetic/antibiotic was added and subsequently diluted serial 2-fold in 0.2% BSA (in 0.01% acetic acid). Then to each well of the first 11 columns 100 μL of mid-log phase bacterial suspension in MHB medium was added to get 2–5 × 10^5^ CFU/mL. Cultures without test peptidomimetics/antibiotics were used as growth control. Uninoculated MHB medium was used as negative control. The microtitre plates were incubated overnight with agitation (180 rpm) at 37 °C. After 16–18 h incubation, absorbance was read at 600 nm using an ELISA plate reader (Molecular Devices, Sunnyvale, CA, USA). The experiments were carried out in duplicate on at least three different days. MIC is defined as the lowest concentration of test agent that completely inhibited bacterial growth.

### MIC determination against MRSA ATCC 33591 under different incubation conditions

An initial inoculum of mid-log phase 10^7^ CFU/mL was used and MIC experiment was performed as described earlier. For serum/plasma stability assay a method as described previously was used with slight modifications^24^. Briefly, 50 μL MHB medium containing desired concentration of test peptidomimetics was incubated with 50 µL mice plasma/fetal bovine serum for 3 h at 37 °C. After incubation, a 10 μL aliquot was taken out for MIC determination as described above. For the experiment, mid-log phase 2–5 × 10^5^ CFU/mL were used as initial inoculum. For the growth control wells, an equivalent amount of plasma/FBS in the medium was also added to evaluate their effect on the viability of cells. A slight decrease in OD_600_ of cells incubated with mice plasma was observed compared to untreated growth control in MHB alone. The experiments were repeated on two different days and reproducible MIC values were observed.

### Hemolysis assay

For the experiment, a previously defined procedure was used with slight modifications^67,68^. Briefly, blood was withdrawn from mice on the day of the experiment. After washing red blood cells (RBC) twice with phosphate buffer saline (PBS: 35 mM sodium phosphate, 150 mM NaCl, pH 7.4), a 4% v/v suspension of RBC was prepared in the same buffer. In a 96-well plate, serial two-fold dilutions of desired concentrations of peptidomimetics (in PBS) were prepared (100 μL) in duplicate. To each well 100 μL of 4% v/v RBC suspension was added. The plates were incubated at 37 °C for 1 h. After 1 h, the plates were centrifuged at 1500 rpm for 10 min and 20 μL of the supernatant was transferred to a fresh 96-well plate containing 80 μL PBS. The hemoglobin release was quantified by measuring OD_414_ in an ELISA plate reader (Molecular Devices, Sunnyvale, CA, USA). Hemoglobin release from 0.1% Triton X-100 treated RBC was set as 100% lysis (A_max_) while as negative control RBC suspended in PBS alone was used (A_PBS_). Percentage hemolysis was calculated using the following equation:

1 $$ \% \,{\rm{hemolysis}}=[({{\rm{A}}}_{{\rm{sample}}}-{{\rm{A}}}_{{\rm{PBS}}})/({{\rm{A}}}_{{\rm{\max }}}-{{\rm{A}}}_{{\rm{PBS}}})]\times 100$$

For the experiment CPCSEA (Committee for the Purpose of Control and Supervision of Experiments on Animals) and Institutional Animal Ethics Committee (IAEC-02/2014) guidelines of JNU, New Delhi, India were followed.

### Cytotoxicity assay

For the active peptidomimetics cytotoxicity was measured against 3T3 murine fibroblast cell line using a previously defined MTT assay with slight modifications^26^. Briefly, the cells were grown to over 75% confluence in DMEM supplemented with 10% FBS and antibiotics (1 × Anti-anti) at 37 °C in 5% CO_2_ atmosphere. The cells were transferred to a 24-well plate to a final count of ~8 × 10^4^ cells/well. After 24 h, the medium was aspirated and desired concentrations of peptidomimetics (10× and 20× their respective MIC in DMEM with 10% FBS) were added to each well. The plates were kept for 2 h at 37 °C in 5% CO_2_ incubator. After 2 h, the cells were washed once with 1 mL PBS. Then 1 mL MTT (0.1 mg/mL) was added to each well in dark and incubated for 2 h at 37 °C in 5% CO_2_. After 2 h, 200 μL of DMSO was added to the wells to dissolve the formazan crystals formed and the plates were kept for 5 min in the incubator at 37 °C. 100 μL of the suspension was transferred to a fresh 96-well plate and absorbance (A_sample_) was measured at 570 nm in an ELISA plate reader (Molecular Devices, Sunnyvale, CA, USA). The wells with DMSO treatment were set as untreated control (A_untreated control_). The assay was done in triplicate on two different days. The % survival was calculated using the following equation:

2 $$ \% \,{\rm{survival}}=[{{\rm{A}}}_{{\rm{sample}}}/{{\rm{A}}}_{{\rm{untreatedcontrol}}}]\times 100$$

### Membrane depolarization assay

The cytoplasmic membrane depolarization in MRSA ATCC 33591 strain was measured using membrane potential sensitive dye 3,3′-dipropylthiacarbocyanine iodide (DiSC_3_(5)) with a protocol described previously with slight modifications^23,48^. Briefly, exponential phase cells grown in MHB were harvested by centrifugation, washed and re-suspended in respiration buffer (5 mM HEPES, 20 mM glucose, pH 7.2) to 2.5 × 10^6^ CFU/mL. The cells were divided into 1 mL aliquots and incubated with 3.6 μM of DiSC_3_(5) (in dark) for 30 min to get steady baseline fluorescence intensity. The dye-loaded cells (800 μL) were then treated with peptidomimetics at respective 1× and 4× MIC concentrations (2–20 μL) and consequent fluorescence increase after 2 min of addition was measured in a Shimadzu RF-5301 PC spectrofluorimeter. As controls IL, melittin and VAN were also assayed under identical conditions. The samples were excited at 622 nm and emission was recorded between 650–700 nm with 3 nm slit width for both excitation and emission. The temperature was maintained at 37 °C throughout the experiment. Depolarization kinetics was measured at 4 × MIC of test agents for 5 min. The experiment was repeated on two different days and for Fig. 3 panel (A) mean ± S.D. data for % depolarization relative to melittin control (10 μM) set as 100% is presented here. For panel (B) representative data is shown here. The viability of dye-loaded cells corresponding to depolarization experiment at 4 × MIC concentration was also measured by plating the diluted aliquots onto BHI agar plates.

### Membrane permeabilization assay

To assess the effect of designed peptidomimetics on membrane integrity of MRSA ATCC 33591 strain a calcein-AM dye release experiment was performed using the protocol described previously with slight modifications^51^. Briefly, exponential phase MRSA ATCC 33591 culture was prepared in MHB medium as mentioned earlier and adjusted to OD_600_ = 1 (~10^9^ CFU/mL) in PBS (10 mM sodium phosphate, 150 mM NaCl, pH 7.4). Thereafter, the cells were incubated at 37 °C with calcein-AM (2 μg/mL) in 10% MHB medium for 2 h. The calcein-loaded cells were washed once and re-suspended in PBS to OD_600_ = 0.5 (~10^8^ CFU/mL). The cells were diluted 100× in PBS to get 3–5 × 10^5^ CFU/mL and were incubated with different concentrations of test agents at 37 °C in dark for 2 h. Subsequently, calcein fluorescence was measured via flow cytometry (Becton Dickinson (BD) FACS verse, San Jose, CA) with excitation and emission wavelengths of 488 nm and 530 ± 30 nm, respectively. For each sample, 10,000 cells were acquired. As a positive control, melittin (10 μM) was assayed under identical conditions. The experiment was repeated on two different days and mean ± S.D. is presented here. Corresponding to leakage a viability assay at 4 × MIC of respective test agents was also performed by incubating samples for 2 h at 37 °C and plating the diluted aliquots on BHI agar plates.

### Scanning electron microscopy (SEM)

For the experiment, a method as described previously was used with slight modifications^69^. In brief, exponential phase MRSA ATCC 33591 culture was prepared as mentioned earlier and adjusted to OD_600_ = 1 (~10^9^ CFU/mL) in PBS (10 mM sodium phosphate, 150 mM NaCl, pH 7.4). Different aliquots (3.5 mL) of this suspension (in 10% MHB) were incubated with the desired concentration of peptidomimetics (10 × MIC) for 4 h at 37 °C with shaking. Since for microscopy higher inoculum is required (10^9^ CFU/mL) therefore, we used 10 × MIC concentrations of designed peptidomimetics. The growth control was untreated bacterial cells. After 4 h, the aliquots were centrifuged and the pellet was washed with PBS twice. The cells were fixed with 2.5% glutaraldehyde at 4 °C overnight. After fixation, the cells were centrifuged and washed with PBS twice. The cells were then dehydrated and using an automatic sputter coater (Polaron OM-SC7640) were coated with 20 nm gold particles. Thereafter, the samples were viewed via scanning electron microscopy (EVO 40, Carl Zeiss, Germany). The experiment was repeated on two different days and similar data was obtained. Representative data are presented here.

### Evaluation of resistance development in MRSA ATCC 33591 strain over 17 serial passages

For the experiment, a method as described previously was used with slight modifications^24^. Briefly, for the experiment, on day 1 the initial MICs of all test agents were determined using routine MIC experiment protocol as described earlier. Next day, bacterial cells (100 μL) from the wells with concentrations 2–4 fold less than the MIC value for each test agent were used to set up fresh mid-log phase cultures in MHB medium for setting up the MIC plate. This was repeated for a total of 17 sub-lethal treatments with each antimicrobial agent. For every passage, corresponding glycerol stocks (15% v/v) were made for each culture and were kept at −80 °C for future use. For this experiment MIC was defined as the lowest concentration of agent which showed no visible growth of bacterial cells.

### Bacterial killing kinetics assay

The kinetics of bacterial killing was determined against MRSA ATCC 33591 strain using drop plate method as described previously with slight modifications^51,67,70,71^. The experiment was performed against exponential phase MRSA in normal medium and stationary phase MRSA in nutrient-depleted medium. For the preparation of nutrient-depleted medium, the primary MRSA ATCC 33591 culture in MHB medium was allowed to grow for 18 h at 37 °C and the cells were removed by centrifugation. The cell pellet was used for stationary phase culture and the spent medium supernatant was used as a nutrient-depleted medium. For obtaining exponential phase cells, MRSA was grown overnight in MHB and 100 μL suspension was used to inoculate 10 mL fresh MHB medium. The cells were incubated at 37 °C for 4–6 h to reach exponential phase.

For the experiments, both exponential and stationary phase cells were re-suspended separately in normal or nutrient-depleted mediums to OD_600_ = 0.5 (~10^8^ CFU/mL). A 25 µL aliquot of this suspension was added to 475 μL of respective medium with or without test peptidomimetics/antibiotics in an eppendorf tube. Based on stock concentration, a volume between 2–20 μL of different peptidomimetics/VAN was added to get the desired concentrations. Subsequently, the samples were incubated at 37 °C. Aliquots of the samples were removed at fixed time intervals and were serially diluted (10-folds) in PBS (10 mM sodium phosphate, 150 mM NaCl, pH 7.3). For plating of samples the BHI agar plate was divided into 9 equal sections and for each dilution three drops of 15 μL bacterial suspension was placed as a drop in each section. The agar plates were incubated at 37 °C for 16–18 h and the colonies were counted (dilutions having colonies in the range of 10–100 were counted). The data was plotted as log CFU/mL vs. time. As a control standard antibiotic VAN was also included in the experiment. The limit of detection for the experiments was 66.7 CFU/mL at 1 × dilution. The experiment was repeated on two different days and similar data was obtained. Representative data from one set is presented here.

### Biofilm formation inhibition assay

For the experiment, a protocol as described previously was used with slight modifications^24^. Briefly, desired concentrations of the test agents in PBS were added to the wells of a 96-well plate (100 μL). Thereafter, overnight grown stationary phase MRSA ATCC 33591 culture (~4 × 10^5^ CFU/mL) in TSB medium (supplemented with 0.25% glucose and 0.5% NaCl) was added to the 96-well plates (100 μL). The plates were incubated at 37 °C without shaking for 24 h. After 24 h, the used medium was discarded and the biofilms formed were washed with PBS once. Subsequently, CV and AB assays were performed to quantify biomass and viability respectively, as described below:

For CV assay after washing the biofilms, the plates were heat fixed at 60 °C for 1 h and 0.1% aqueous CV solution (200 μL) was added to the wells. After 15 min incubation, excess CV was washed off and biomass adhered CV was dissolved in 33% glacial acetic acid (200 μL). The plates were kept at room temperature for 15 min. The contents of the wells were mixed thoroughly and were transferred to a fresh plate while being diluted 5 × in 33% glacial acetic acid. To quantify CV associated biomass, the absorbance was read at 570 nm.

For AB assay, after washing the treated biofilms, a solution of 10% AB reagent in TSB medium (200 μL) was added to the wells of the plates. The plates were sonicated briefly in a bath sonicator (5 min) and kept at room temperature for 30 min in dark. After 30 min, fluorescence was measured by exciting the samples at 550 nm and collecting emission at 590 nm.

The experiments were repeated on two different days and on each day 6 wells were used for a single treatment. Mean ± S.E. for both CV assay and AB assay is presented here. Corresponding to CV and AB assay the viability of cells upon treatment was also evaluated. Towards this 200 μL PBS was added to washed and treated wells of the 96-well plate and the contents were scraped thoroughly. The resulting bacterial suspension was sonicated briefly, diluted in PBS and plated on BHI agar plates. The agar plates were incubated at 37 °C for 16–24 h and CFUs were counted. Mean ± S.E. data from two independent experiments performed on two different days is shown here.

### Evaluation of efficacy against 48 h mature MRSA biofilms

For the experiment, a protocol as described previously was used with slight modifications^24,72^. Briefly, stationary phase MRSA ATCC 33591 culture ~10^5^ CFU/mL in TSB medium (supplemented with 0.25% glucose and 0.5% NaCl) was added to the well of an 8-well chamber slide (200 μL). The slide was incubated at 37 °C without shaking for 24 h. After 24 h, the medium was discarded and the biofilm was washed with PBS once (10 mM sodium phosphate, 150 mM NaCl, pH 7.3). The desired concentration of peptidomimetics or VAN (20 × MIC) in TSB medium (200 μL) were added to the wells and the chamber slides were further incubated at 37 °C for 24 h without shaking. After 48 h (24 h + 24 h), the planktonic cells were removed and the biofilms were washed with PBS once. To the wells, 200 μL of PBS containing BacLight Live/Dead viability stain [SYTO9 (green fluorescence) and propidium iodide (PI) (red fluorescence) in equal volume] was added and the chamber slides were incubated at room temperature for 15 min in dark. After 15 min, the stain was removed and biofilms were washed with PBS once. To fix the biofilms, 200 μL of neutral buffered formalin (10% in PBS) was added to each well and the slides were kept at room temperature in dark for 30 min. After fixation, the biofilms were washed once, the chamber was removed and a cover slip was placed over the slide. The biofilms were viewed with an Olympus FluoView^TM^ FV1000 confocal laser scanning microscope (2D and z-stacking). For each sample, at least four different regions were scanned. For each sample, the biofilms thickness was also determined using z-scanning confocal microscopy. For the treated samples, the regions where biofilms were visible were imaged and the experiments were repeated on three different days. Representative data is presented here.

### Evaluation of cell viability against biofilm embedded cells

Corresponding to the confocal laser scanning microscopy experiment viability of 48 h mature biofilm embedded cells was also quantified by running parallel chamber slides with identical treatments (20 × MIC). For enumeration of cells after 48 h incubation, the medium was aspirated and the biofilms were washed with PBS once. Then 200 μL PBS was added to each well of the chamber slide and the chambers were thoroughly scraped with a pipette tip. The bacterial suspension obtained was plated on BHI agar after dilution in PBS as described above. The resulting CFU/mL from two different days were counted and plotted as mean ± S.D.

### Statistical analysis

For calculation of statistical significance GraphPad Prism 5 software was used and one-way analysis of variance (ANOVA) with post hoc Bonferroni’s test was applied where ‘p’ value of < 0.05 was considered significant.

### Data Availability

Most of the experimental data acquired/analyzed during this study have been included in this published article and also provided as ESI. Information on rest of the data can be obtained from the corresponding author(s) on request.

## Electronic supplementary material

Supplementary Information
