# Supplementary material for: Novel Miniature Membrane Active Lipopeptidomimetics against Planktonic and Biofilm Embedded Methicillin-Resistant Staphylococcus aureus
Source: Sci Rep. 2018 Jan 18;8:1021. doi: 10.1038/s41598-017-17234-z (PMC5773577; doi:10.1038/s41598-017-17234-z)
Supplement: Supplementary file 1 — Supplementary Information [file 41598_2017_17234_MOESM1_ESM.pdf]

ELECTRONIC SUPPLEMENTARY INFORMATION (ESI)

Novel Miniature Membrane Active Lipopeptidomimetics against Planktonic and Biofilm Embedded Methicillin-Resistant *Staphylococcus aureus*

Seema Joshi[a,*], Sana Mumtaz[a], Jyotsna Singh[a], Santosh Pasha[b] & Kasturi Mukhopadhyay[a,*]

Antimicrobial Research Laboratory, School of Environmental Sciences, Jawaharlal Nehru University, New Delhi-110067, India[a]; Peptide Research Laboratory, CSIR-Institute of Genomics and Integrative Biology, Mall Road, New Delhi-110007, India[b]

***Corresponding author(s):** joshi.seema25@gmail.com or kasturim@mail.jnu.ac.in

Table of Contents

Fig. S1: Reverse phase-HPLC chromatograms for peptidomimetics S-1 to S-9.

Fig. S2: Electrospray ionization-mass spectrometry data for peptidomimetics S-1 to S-9.

Fig. S3: Membrane depolarization mechanism using $DiSC_3(5)$ dye.

Fig. S4: Cell viability corresponding to membrane depolarization experiment.

Fig. S5: Cell viability corresponding to calcein leakage experiment.

Fig. S6: Comparative DNA binding ability of designed peptidomimetics.

Fig. S1. Representative RP-HPLC chromatograms of peptidomimetics S-1 to S-9 (absorbance at 220 nm). For the HPLC analysis a linear gradient of acetonitrile (0.1% TFA) from 10 to 90% was run for 45 min, where, water (0.1 % TFA) was the second solvent used for chromatography.

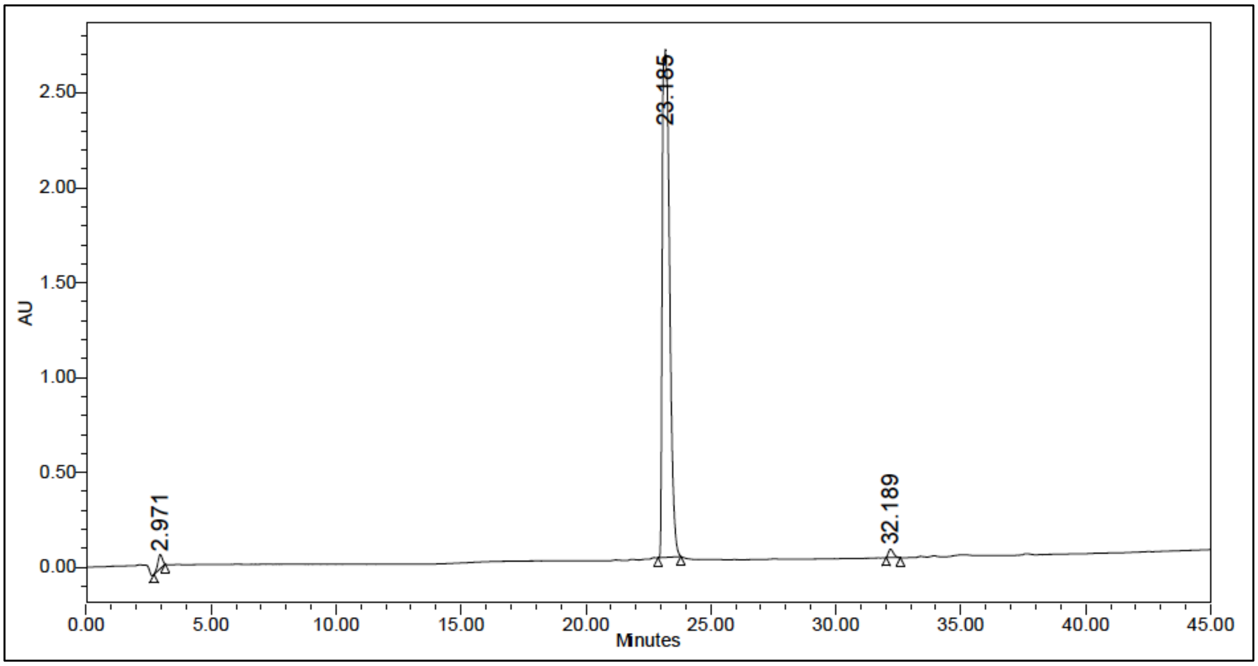

S-1

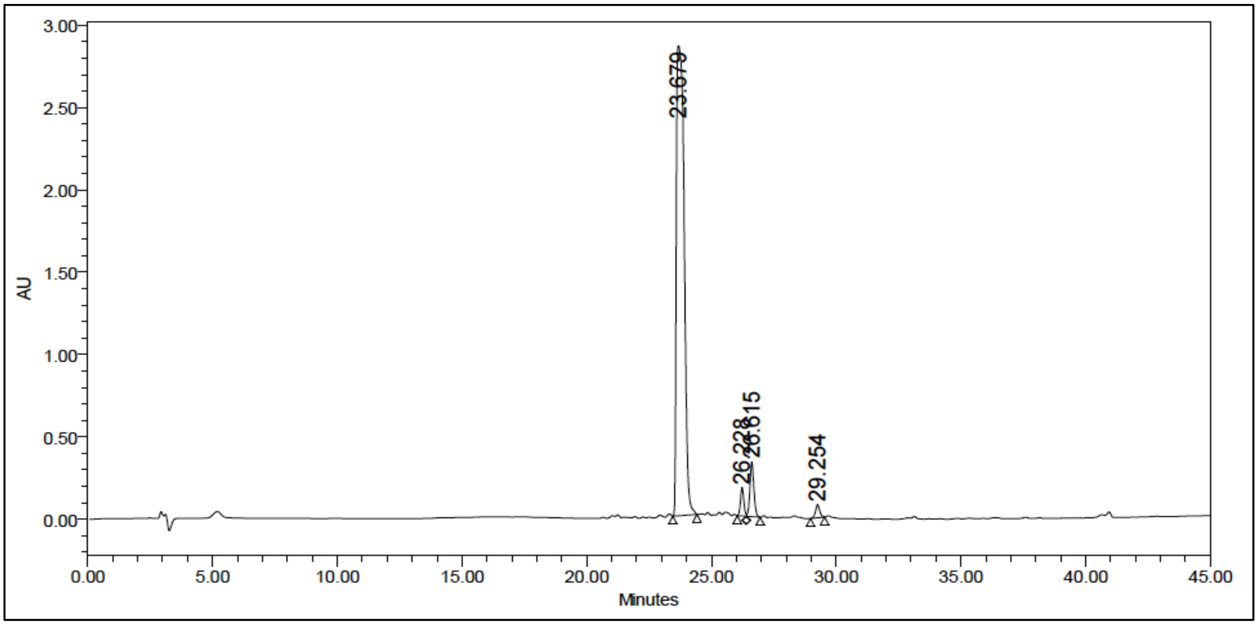

S-2

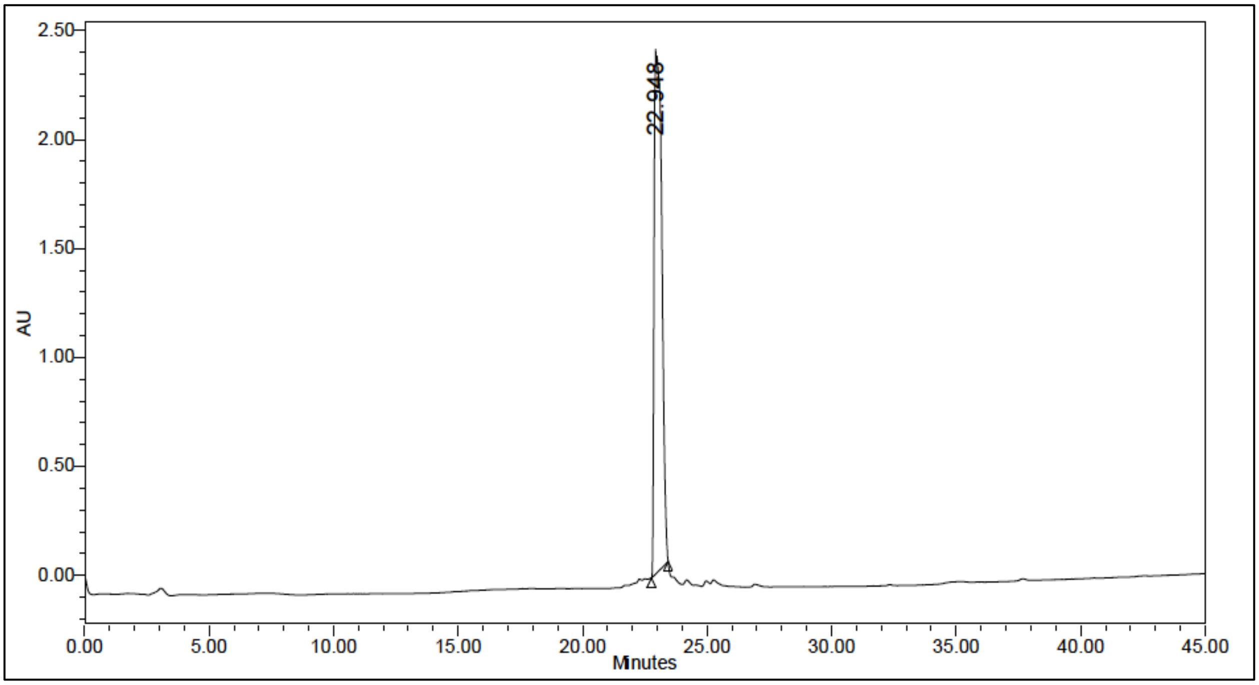
22.948
AU
Minutes
2.50
2.00
1.50
1.00
0.50
0.00
0.00
5.00
10.00
15.00
20.00
25.00
30.00
35.00
40.00
45.00

S-3

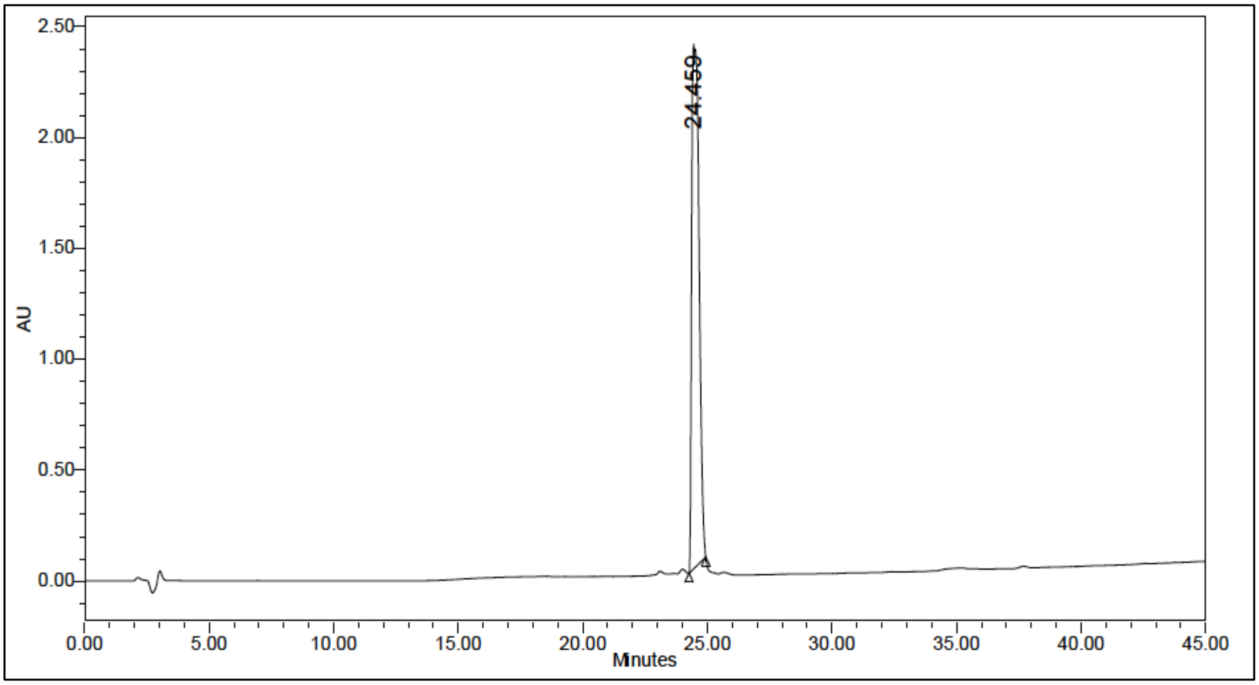
24.459
AU
Minutes
2.50
2.00
1.50
1.00
0.50
0.00
0.00
5.00
10.00
15.00
20.00
25.00
30.00
35.00
40.00
45.00

S-4

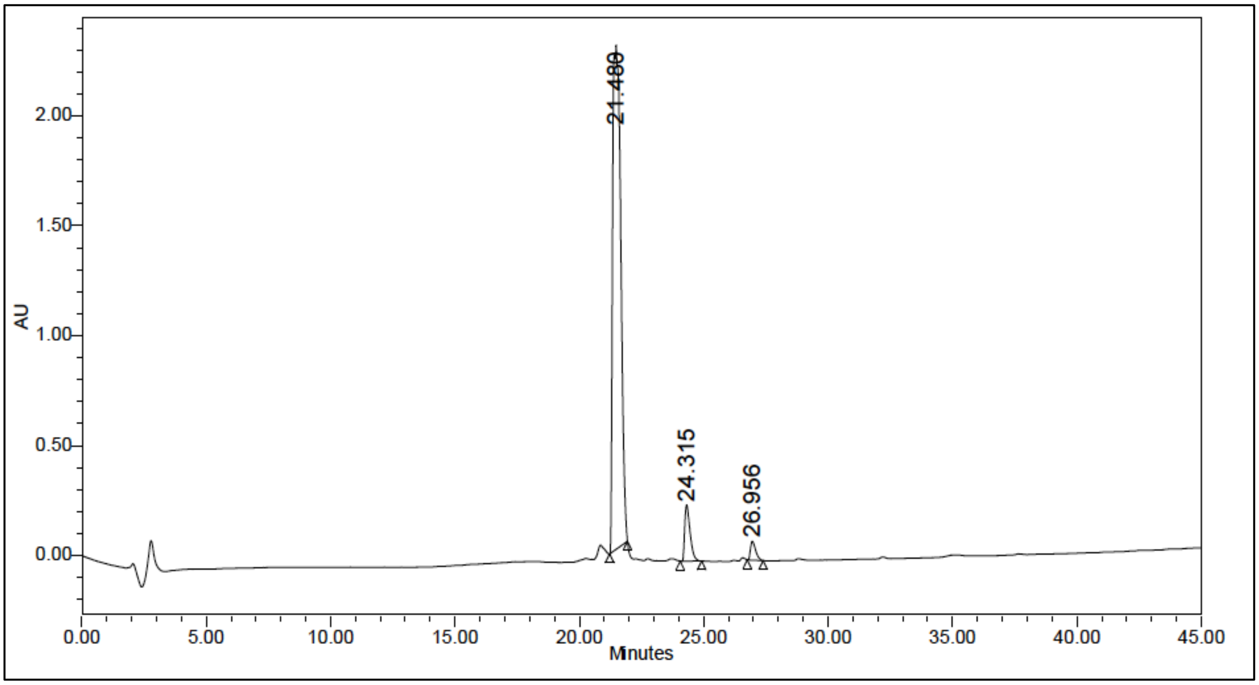

S-5

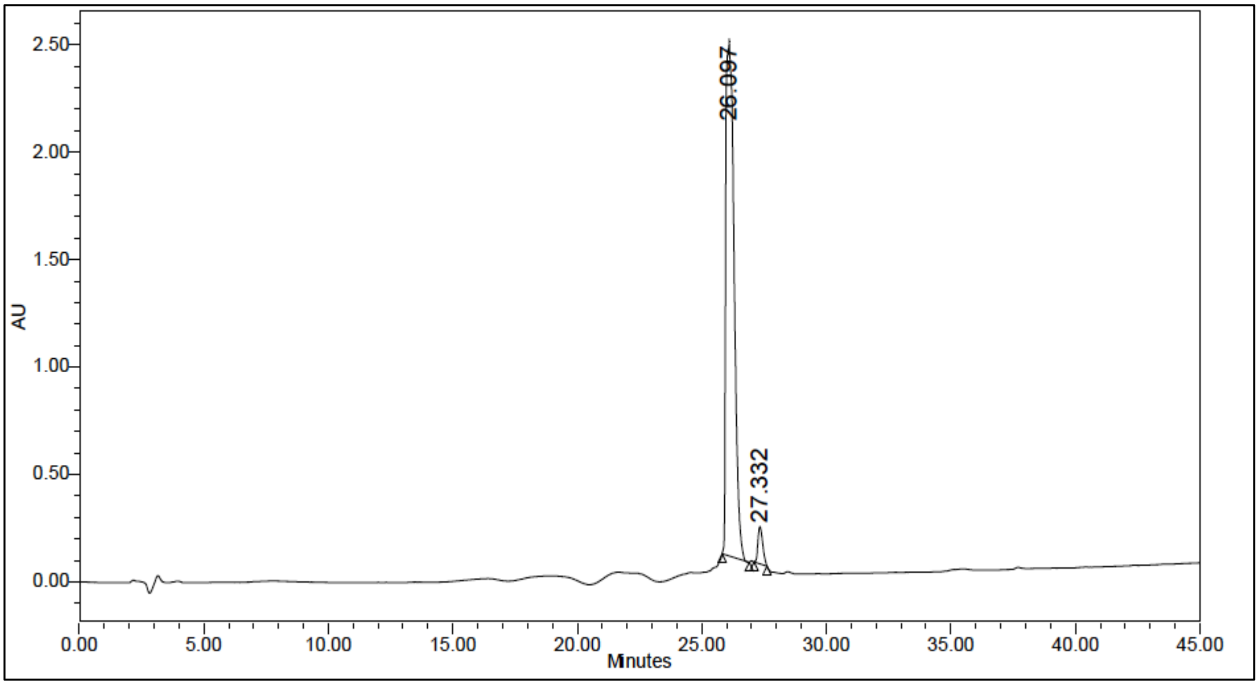

S-6

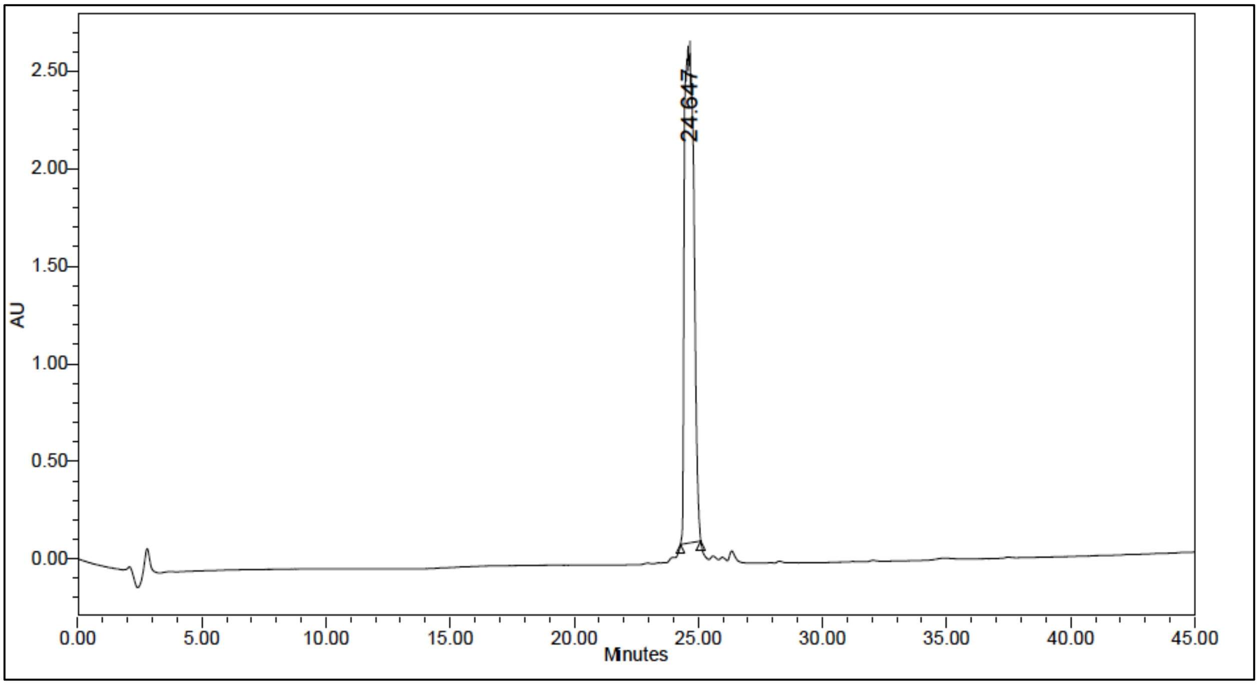
24.647
AU
Minutes
0.00
0.50
1.00
1.50
2.00
2.50
5.00
10.00
15.00
20.00
25.00
30.00
35.00
40.00
45.00

S-7

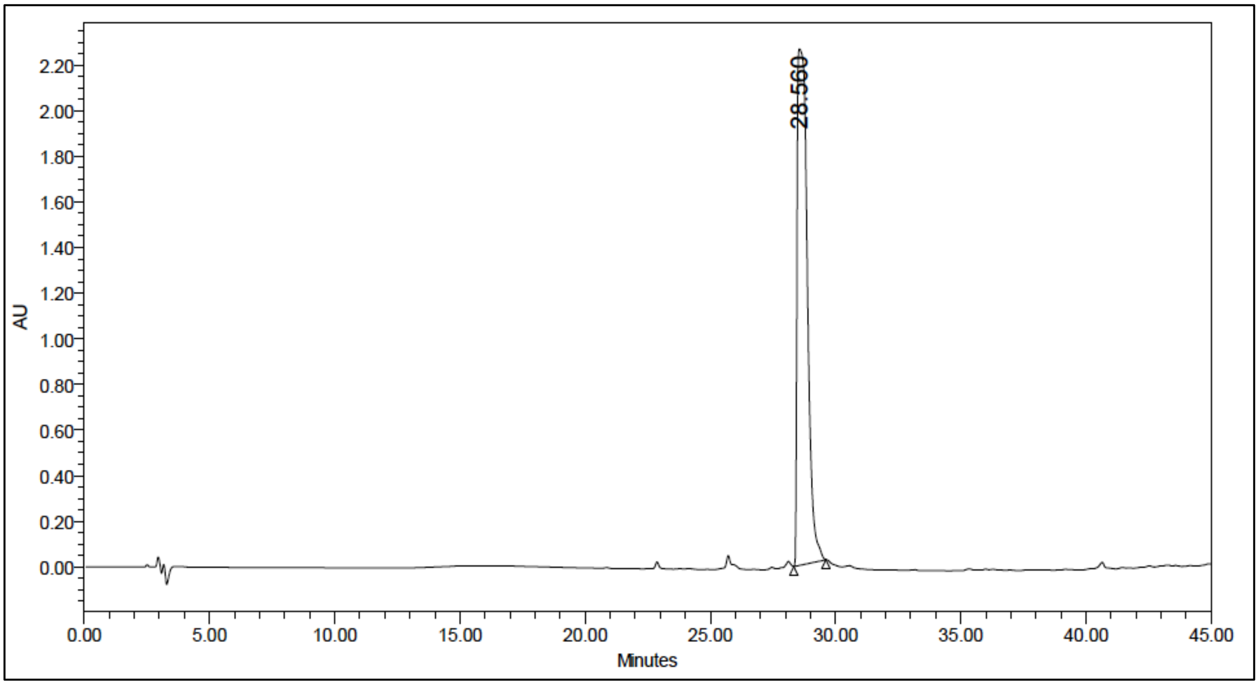
28.560
AU
Minutes
0.00
0.20
0.40
0.60
0.80
1.00
1.20
1.40
1.60
1.80
2.00
2.20
5.00
10.00
15.00
20.00
25.00
30.00
35.00
40.00
45.00

S-8

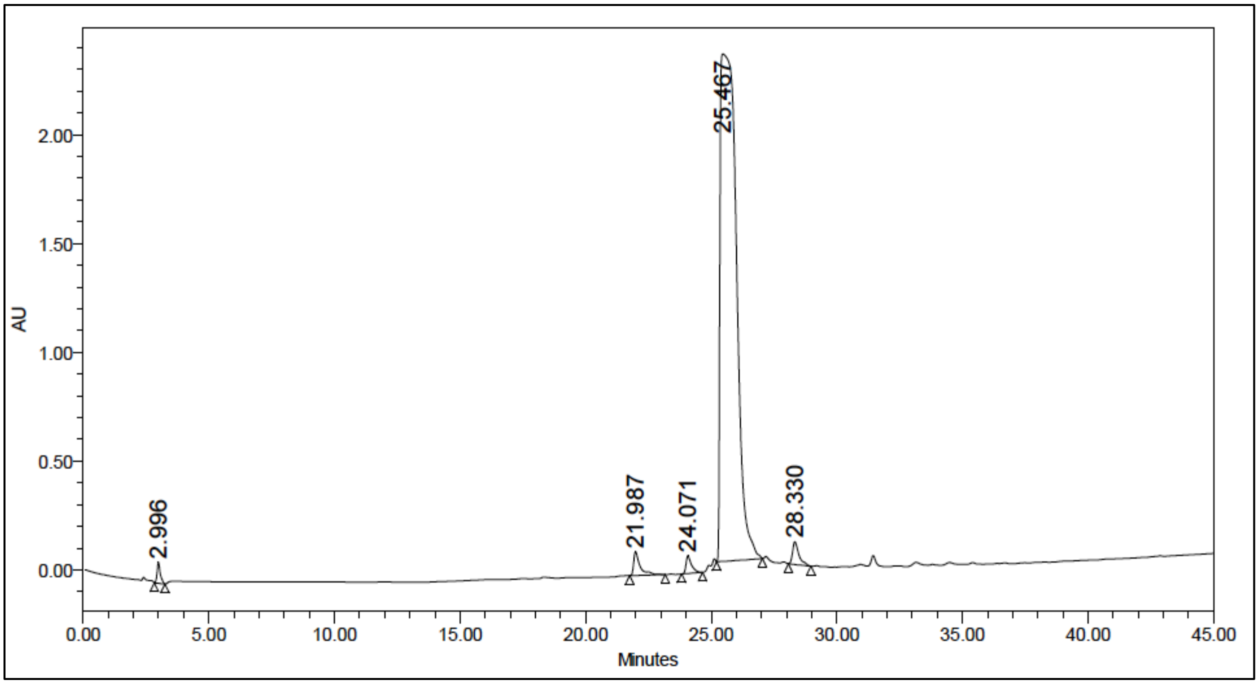

S-9

Fig. S2. Representative electrospray ionization-mass spectrometry data for peptidomimetics S-1 to S-9. The data was acquired on WATERS SYNAPT G2 mass spectrometer (LockSpray methodology) with Acquity UPLC system.

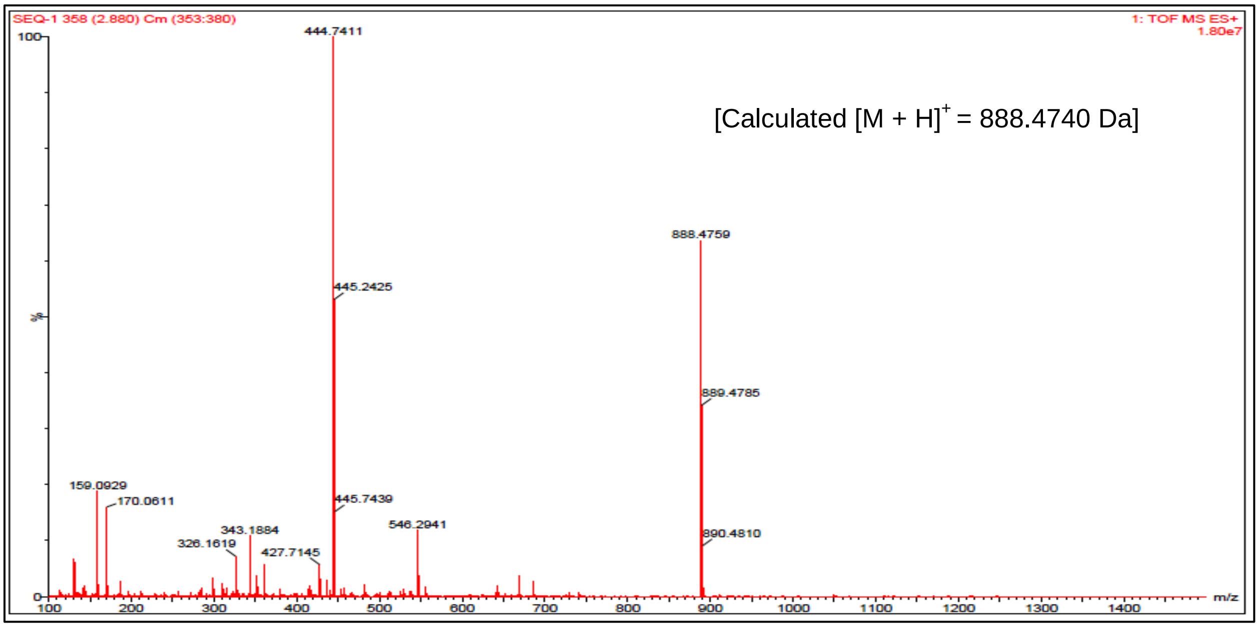

S-1

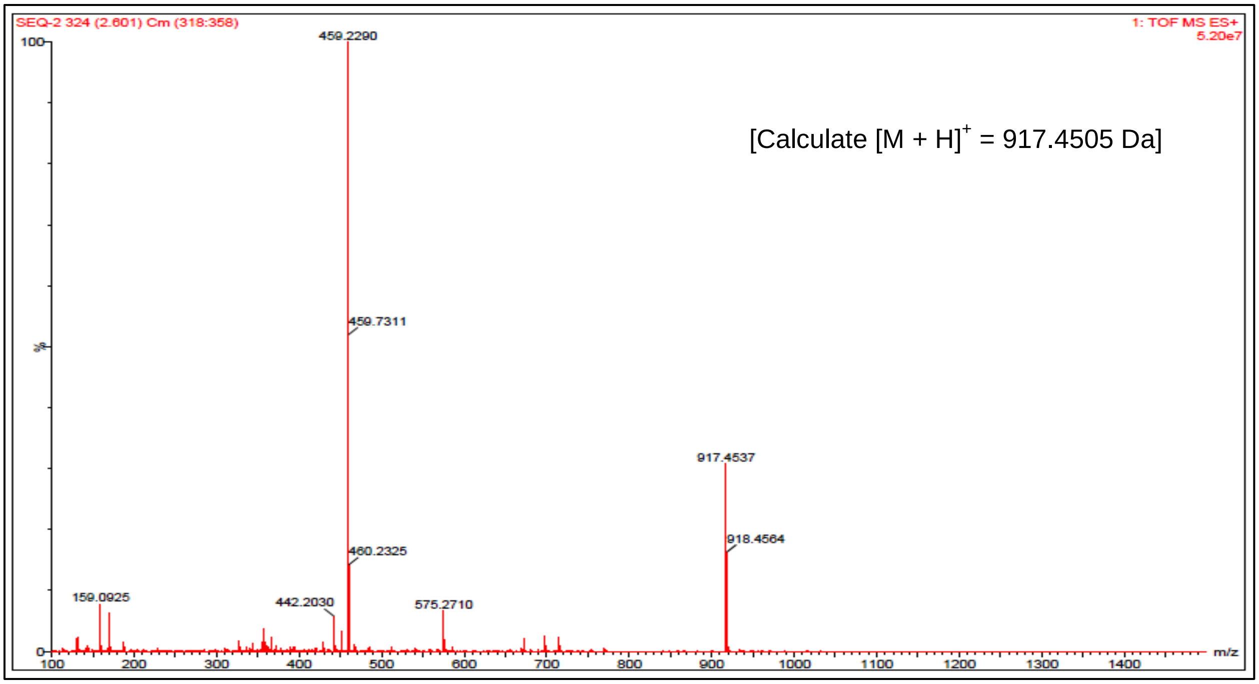
SEQ-2 324 (2.601) Cm (318:358)
1: TOF MS ES+
5.20e7
[Calculate [M + H]+ = 917.4505 Da]
459.2290
459.7311
460.2325
917.4537
918.4564
159.0925
442.2030
575.2710
m/z

S-2

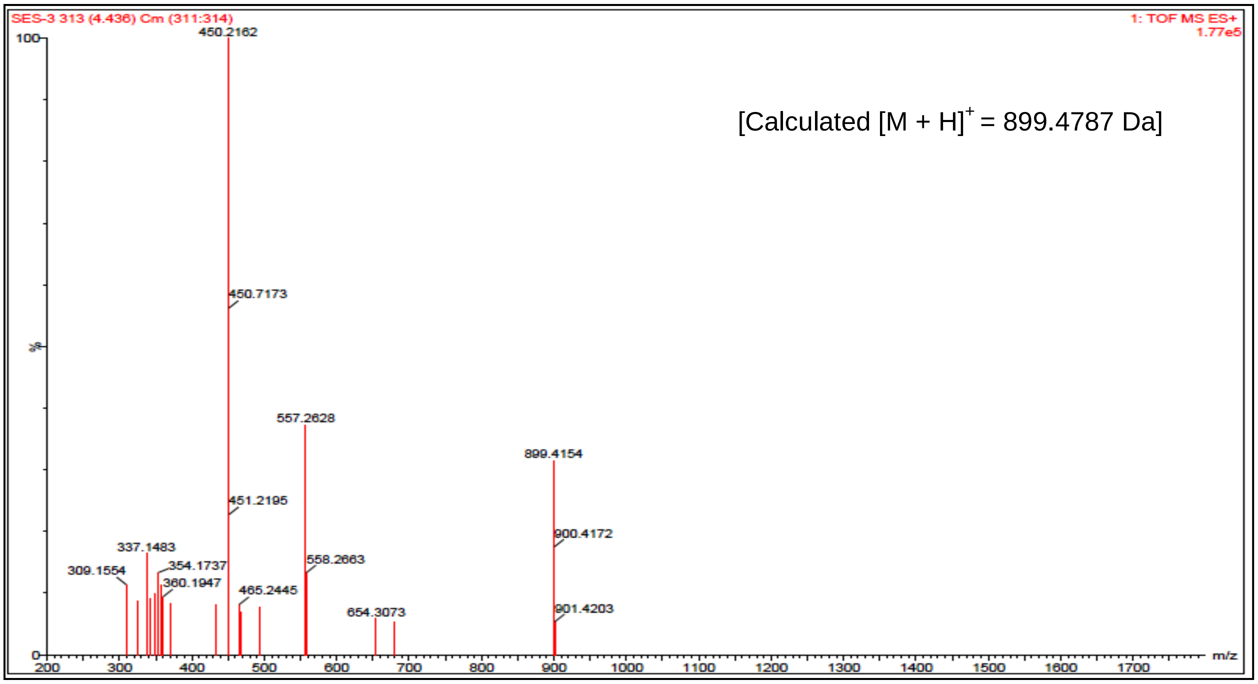
SES-3 313 (4.436) Cm (311:314)
1: TOF MS ES+
1.77e5
[Calculated [M + H]+ = 899.4787 Da]
450.2162
450.7173
557.2628
899.4154
451.2195
900.4172
337.1483
354.1737
309.1554
360.1947
558.2663
465.2445
654.3073
901.4203
m/z

S-3

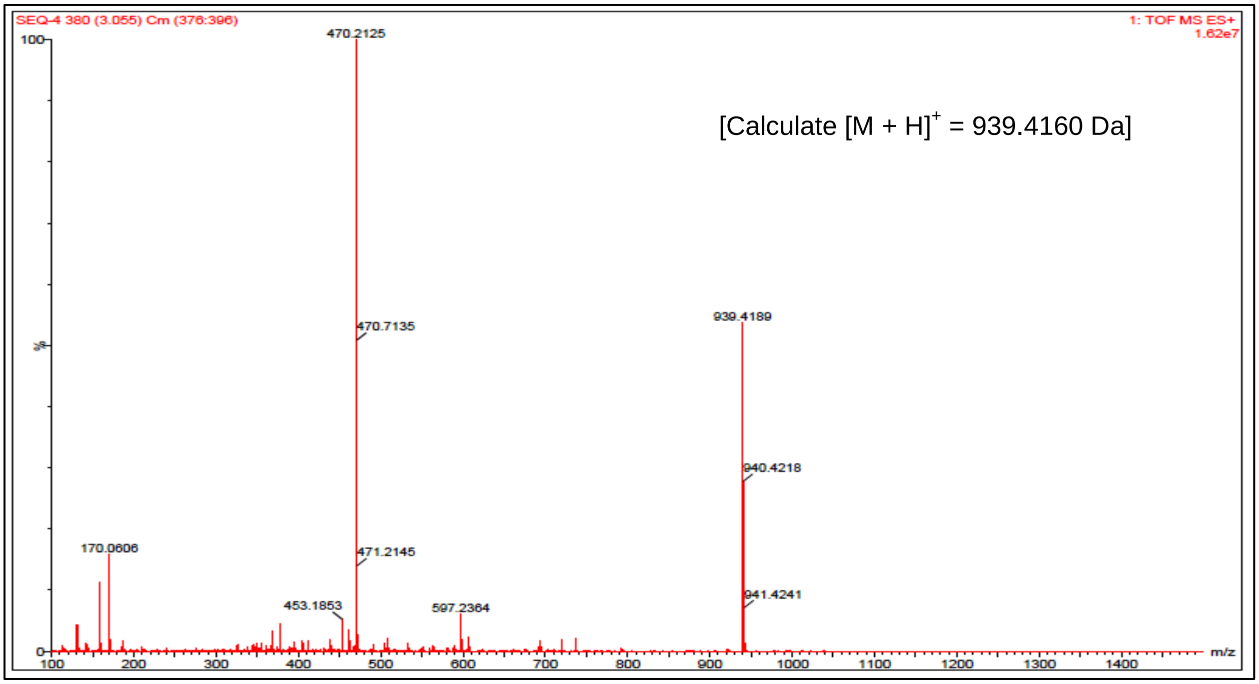
SEQ-4 380 (3.055) Cm (376:396)
1: TOF MS ES+
1.62e7
[Calculate [M + H]+ = 939.4160 Da]
470.2125
470.7135
471.2145
939.4189
940.4218
941.4241
170.0606
453.1853
597.2364
100
%
0
m/z
100
200
300
400
500
600
700
800
900
1000
1100
1200
1300
1400

S-4

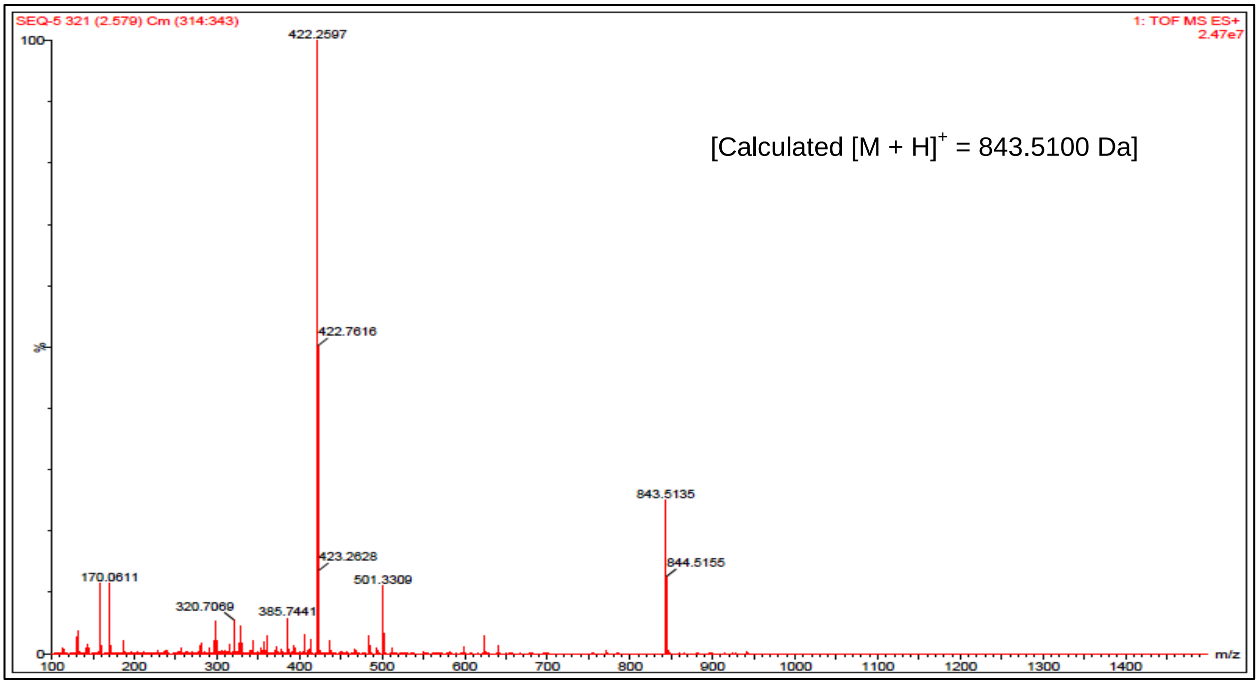
SEQ-5 321 (2.579) Cm (314:343)
1: TOF MS ES+
2.47e7
[Calculated [M + H]+ = 843.5100 Da]
422.2597
422.7616
423.2628
843.5135
844.5155
170.0611
320.7069
385.7441
501.3309
100
%
0
m/z
100
200
300
400
500
600
700
800
900
1000
1100
1200
1300
1400

S-5

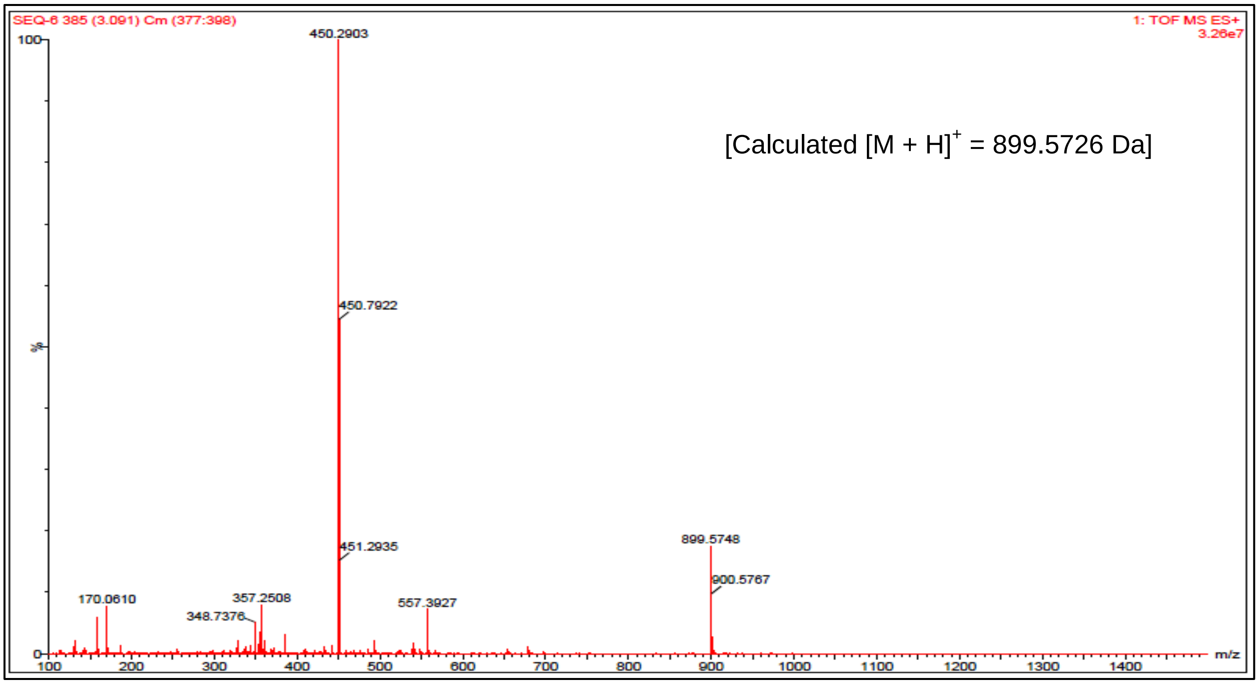
SEQ-6 385 (3.091) Cm (377:398)
1: TOF MS ES+
3.26e7
[Calculated [M + H]+ = 899.5726 Da]
450.2903
450.7922
451.2935
899.5748
900.5767
170.0610
357.2508
348.7376
557.3927
m/z

S-6

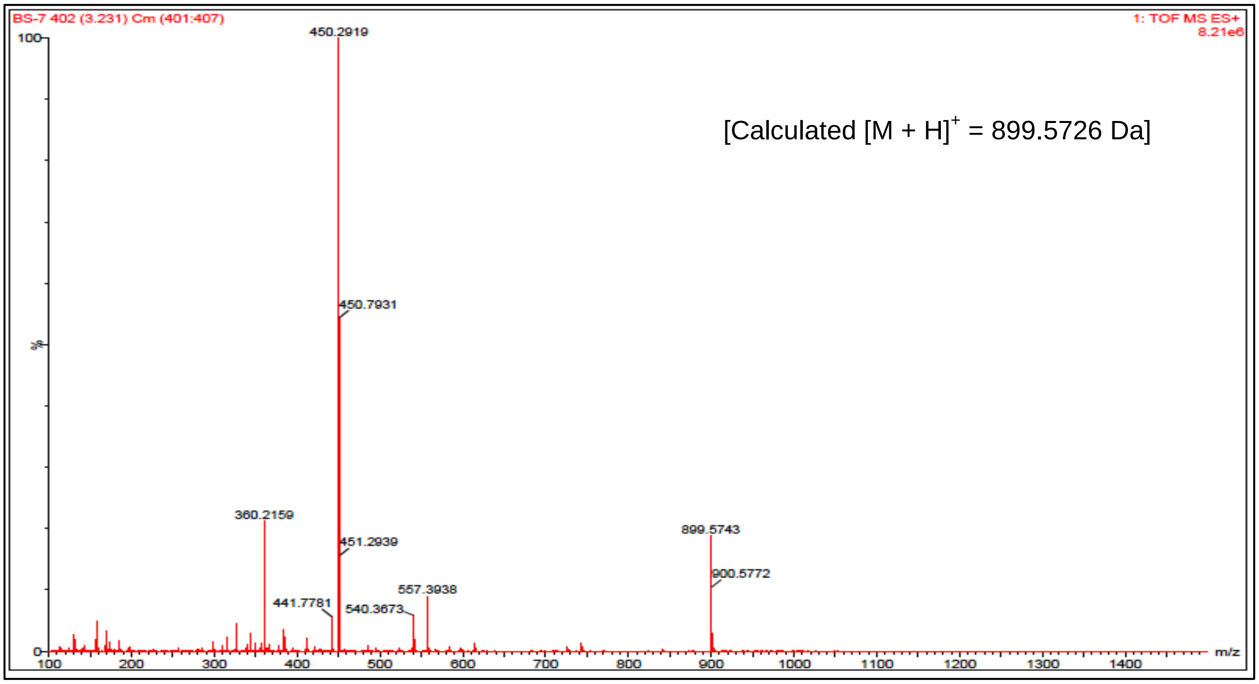
BS-7 402 (3.231) Cm (401:407)
1: TOF MS ES+
8.21e6
[Calculated [M + H]+ = 899.5726 Da]
450.2919
450.7931
360.2159
451.2939
899.5743
900.5772
557.3938
441.7781
540.3673
m/z

S-7

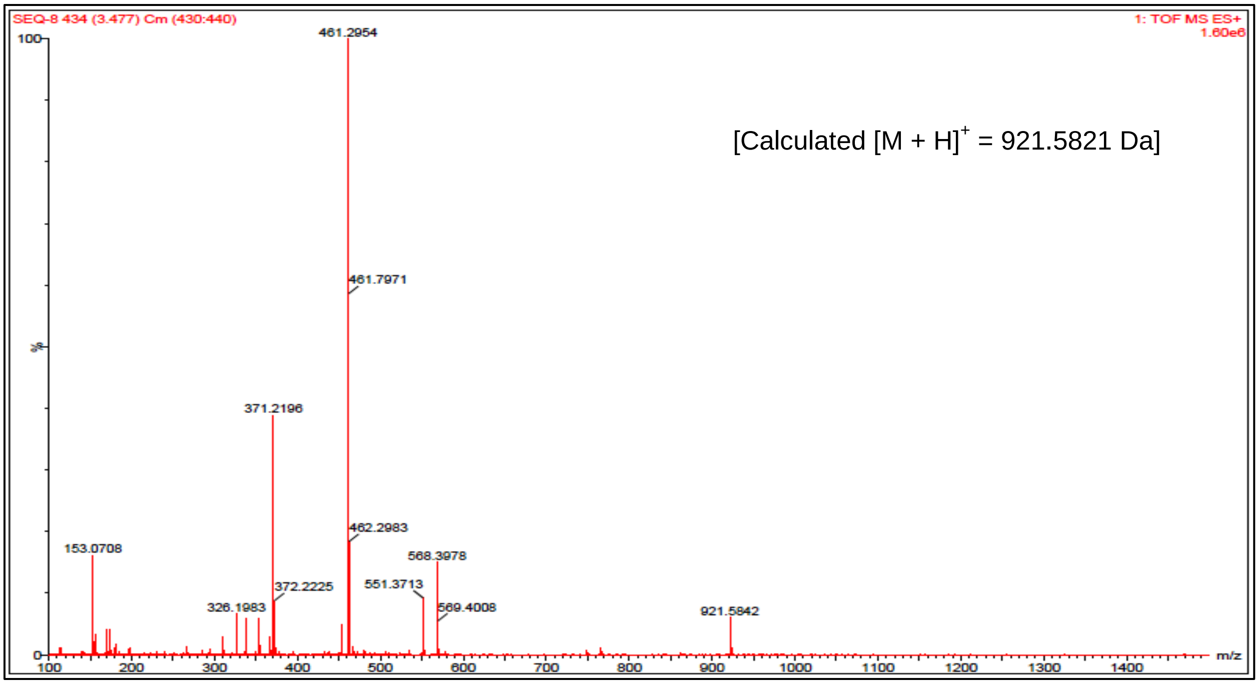
SEQ-8 434 (3.477) Cm (430:440)
1: TOF MS ES+
1.60e6
[Calculated [M + H]+ = 921.5821 Da]
461.2954
461.7971
371.2196
462.2983
153.0708
568.3978
551.3713
372.2225
326.1983
569.4008
921.5842
m/z

S-8

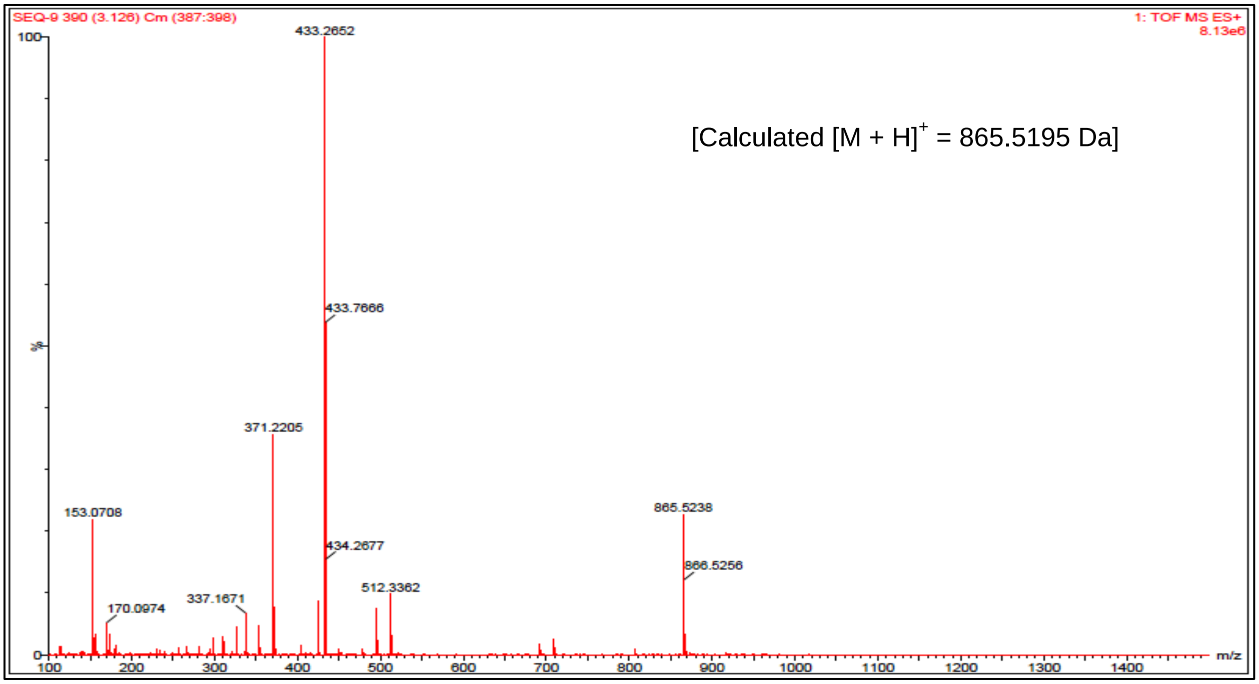
SEQ-9 390 (3.126) Cm (387:398)
1: TOF MS ES+
8.13e6
[Calculated [M + H]+ = 865.5195 Da]
433.2652
433.7666
371.2205
153.0708
865.5238
434.2677
866.5256
512.3362
337.1671
170.0974
m/z

S-9

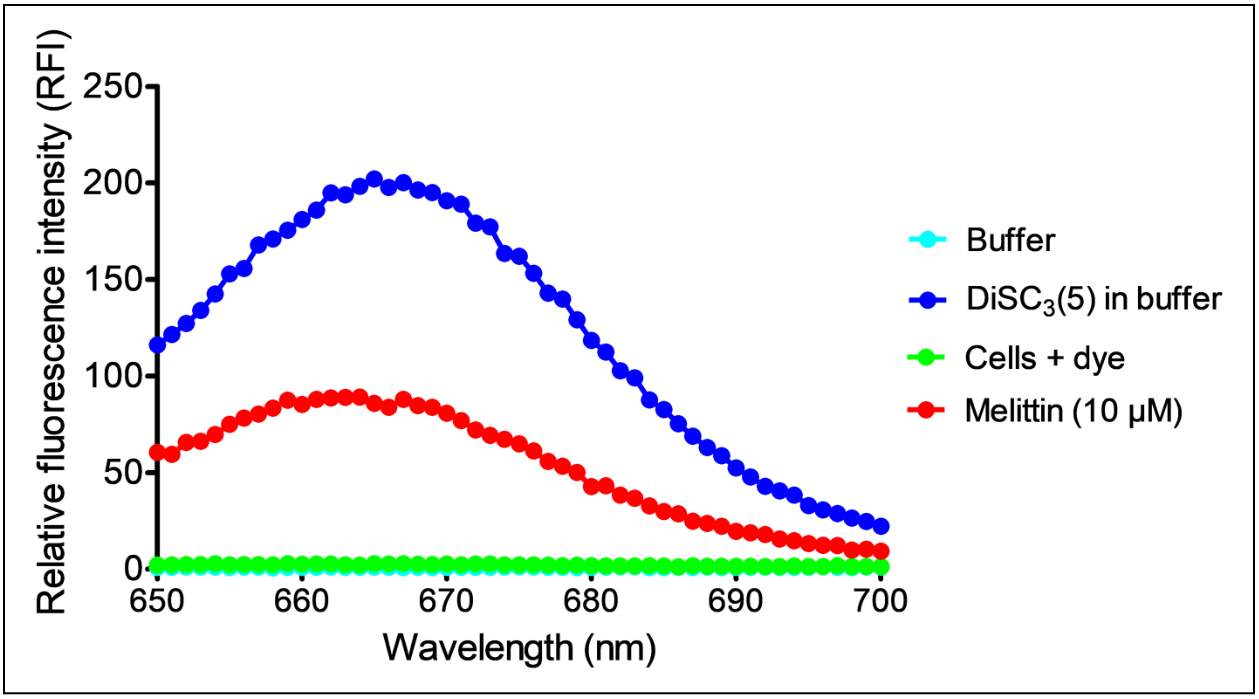

Fig. S3. Membrane depolarization mechanism using DiSC$_3$(5) dye. Fluorescence emission spectrum of buffer (5 mM HEPES, 20 mM glucose, pH 7.2), DiSC$_3$(5) dye in buffer, MRSA cells loaded with DiSC$_3$(5) dye for 30 min and melittin (10 μM) added to fluorescence quenched dye-loaded cells. The fluorescence spectrum was acquired at an excitation wavelength of 622 nm on a Shimadzu RF-5301 PC spectrofluorimeter and emission was recorded between 650-700 nm. Slit width of 3 nm was used for both excitation and emission and the temperature was maintained at 37ºC for the experiment.

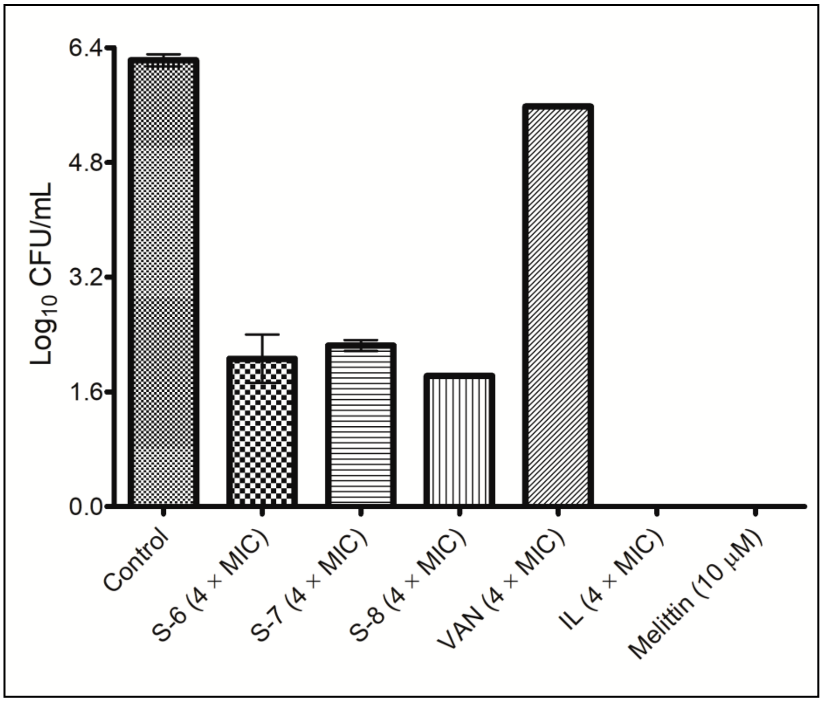

Fig. S4. Cell viability corresponding to membrane depolarization experiment. The dye-loaded cells suspended in HEPES-glucose buffer (5 mM, 20 mM glucose, pH 7.2) were incubated with different test agents for 2 min and subsequently diluted appropriately and plated on BHI agar plates. The plates were incubated at 37 ºC for 16-18 h and colonies were counted. The experiment was repeated on two different days and mean ± S.D. is presented here. The differences between control vs. all treatments were statistically significant ($p<0.05$) except for VAN. The MIC values for test peptidomimetics against MRSA ATCC 33591 strain were as follows: S-6 (11.3 μg/mL), S-7 (11.3 μg/mL), S-8 (1.4 μg/mL), IL (3.2 μg/mL) and VAN (1.4 μg/mL).

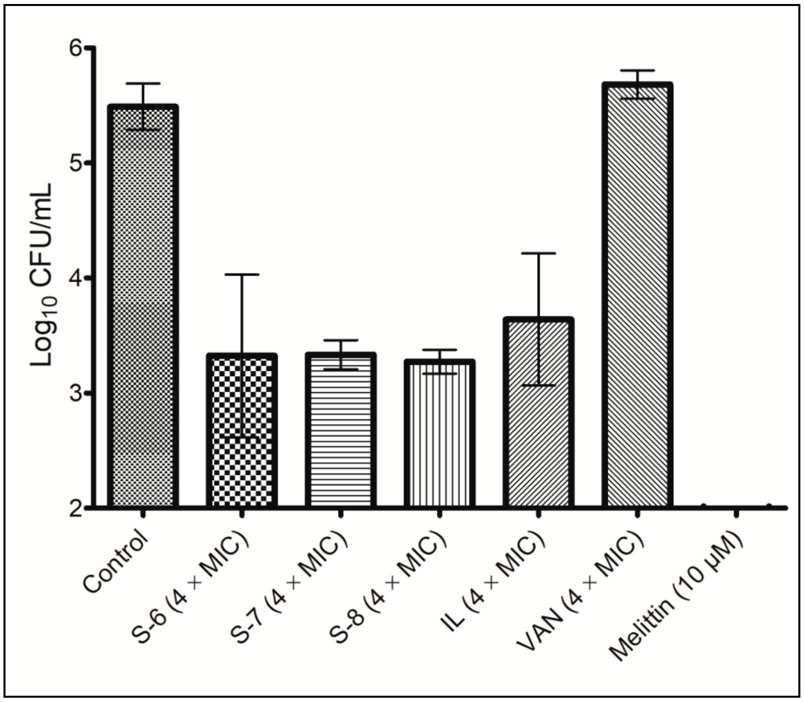

Fig. S5. Cell viability corresponding to calcein dye leakage experiment. The dye-loaded cells suspended in PBS (10 mM, 150 mM NaCl, pH 7.4) were incubated with different test agents for 2 h and subsequently diluted appropriately and plated on BHI agar plates. The plates were incubated at 37 ºC for 16-18 h and colonies were counted. The experiment was repeated on two different days and mean ± S.D. is presented here. The differences between control vs. all treatments were statistically significant ($p<0.05$) except for VAN. The MIC values for test peptidomimetics against MRSA ATCC 33591 strain were as follows: S-6 (11.3 μg/mL), S-7 (11.3 μg/mL), S-8 (1.4 μg/mL), IL (3.2 μg/mL) and VAN (1.4 μg/mL).

DNA gel retardation assay

Methodology: Towards comparing the relative DNA binding ability we performed a gel retardation experiment using a protocol described previously with slight modifications (references 67 and 68 in manuscript). Briefly, different concentrations of test peptidomimetics were incubated with 100 ng plasmid DNA (pBluescript II SK+) for 1 h at room temperature. After incubation, 4 μL native loading buffer was added to the samples and a 20 μL aliquot was subjected to 1% agarose gel electrophoresis in 0.5 × Tris borate-EDTA buffer (45 mM Tris-borate, 1 mM EDTA, pH 8.0). The plasmid DNA was supplied by Agilent Technologies and was used without further purification. The composition of binding buffer was: 5% glycerol, 10 mM Tris-HCl (pH 8.0), 1 mM EDTA, 1 mM dithiothreitol, 20 mM KCl and 50 μg/mL bovine serum albumin (BSA). The composition of native loading buffer was: 10% Ficoll 400, 10 mM Tris-HCl (pH 7.5), 50 mM EDTA, 0.25% bromophenol blue and 0.25% xylene cyanol. The experiment was repeated on two different days and similar results were obtained. Representative data is shown here.

Results: To evaluate if intracellular targeting/DNA binding mechanisms are operative for S-7 and S-8 mediated killing, we performed a relative plasmid DNA binding experiment. Since DNA is negatively charged and the designed peptidomimetics are positively charged, therefore, to rule out electrostatic interactions we also performed gel retardation for S-1 which carries same charge (+3) as S-7 and S-8 but does not show activity against the tested bacterial strains up to 45.4 μg/mL.

The results showed that S-1 completely retarded DNA mobility at 50 μg/mL (Fig. S6). S-7 also completely retarded the movement of plasmid DNA up to 50 μg/mL while at lower concentrations the DNA bands got smeared in the gel reflecting complexation capacity of S-7 at lower concentrations tested. In comparison, S-8 showed retardation in movement of plasmid DNA at 10 μg/mL while IL also caused retardation at 23.8 μg/mL.

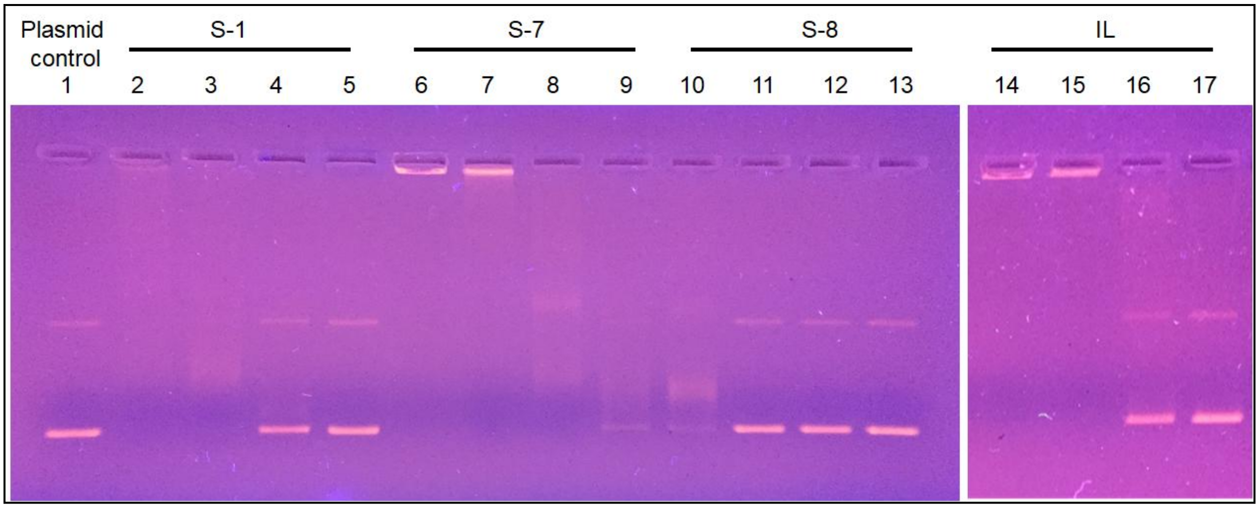

Fig. S6. Comparative DNA binding ability of designed peptidomimetics. The plasmid DNA binding ability of designed peptidomimetics and IL were compared using native 1% agarose gel electrophoresis. Lane 1: plasmid DNA; lane 2: 100 μg/mL S-1; lane 3: 50 μg/mL S-1; lane 4: 25 μg/mL S-1; lane 5: 12.5 μg/mL S-1; lane 6: 100 μg/mL S-7; lane 7: 50 μg/mL S-7; lane 8: 25 μg/mL S-7; lane 9: 12.5 μg/mL S-7; lane 10: 10 μg/mL S-8; lane 11: 5 μg/mL S-8; lane 12: 2.5 μg/mL S-8; lane 13: 1.25 μg/mL S-8; lane 14: 47.6 μg/mL IL; lane 15: 23.8 μg/mL IL; lane 16: 11.9 μg/mL IL and lane 17: 5.9 μg/mL IL. For the assay IL was run under identical experimental conditions in a separate gel.

Thus, whereas S-7 was able to retard DNA movement near its MIC (12.5 μg/mL) for S-8 (10 μg/mL) and IL (23.8 μg/mL) concentrations higher than MIC were obligatory to show retardation in DNA movement.
